# A Strongly Coupled Cluster Heterostructure with Pt–N-Mo Bonding for Durable and Efficient H_2_ Evolution in Anion-Exchange Membrane Water Electrolyzers

**DOI:** 10.1007/s40820-025-01798-x

**Published:** 2025-06-13

**Authors:** Wenbo Zhou, Yichao Huang, Hanqing Cai, Tao Wang, Haitao Li, Chao Zhang, Lianming Zhao, Lulu Chen, Meihong Liao, Zhiqing Tang, Kai Chen, Jing Gu, Wenpei Gao, Zhuangjun Fan, Zhenhai Wen

**Affiliations:** 1 State Key Laboratory of Chemical Safety, Shandong Key Laboratory of Intelligent Energy Materials, School of Materials Science and Engineering, China University of Petroleum (East China), Qingdao, 266580 People’s Republic of China; 2 State Key Laboratory of Metal Matrix Composites, School of Materials Science and Engineering, Future Material Innovation Center, Zhangjiang Institute for Advanced Study, Shanghai Jiao Tong University, Shanghai, 200240 People’s Republic of China; 3 Fujian Institute of Research on the Structure of Matter, Chinese Academy of Science, Institute of Materials, Fuzhou, 350002 People’s Republic of China; 4 School of Mechanical and Electronic Engineering, Qingdao Binhai University, Qingdao, 266555 People’s Republic of China; 5 Institute of Energy, Hefei Comprehensive National Science Center, Hefei, 230051 People’s Republic of China; 6 Department of Chemistry and Biochemistry, San Diego State University, 5500 Campanile Drive, San Diego, CA 92182-1030 USA

**Keywords:** Heterostructures, Polyoxometalates, Electrocatalysis, Hydrogen evolution reaction, Anion-exchange membrane water electrolyzers

## Abstract

Creating strongly coupled heterostructures with favorable catalytic activities is crucial for promoting the performance of catalytic reactions, especially those involve multiple intermediates. Herein, we fabricated a strongly coupled platinum/molybdenum nitrides nanocluster heterostructure on nitrogen-doped reduced graphene oxide (Pt/Mo₂N–NrGO) for alkaline hydrogen evolution reaction. The well-defined Pt-containing Anderson-type polyoxometalates promote strong interfacial Pt–N–Mo bonding in Pt/Mo_2_N–NrGO, which exhibits a remarkably low overpotential, high mass activity, and exceptional long-term durability (> 500 h at 1500 mA cm⁻^2^) in an anion-exchange membrane water electrolyzer (AEMWE). Operando Raman spectroscopy and density functional theory reveal that pronounced electronic coupling at the Pt/Mo₂N cluster interface facilitates the catalytic decomposition of H_2_O through synergistic stabilization of intermediates (Pt–H* and Mo-OH*), thereby enhancing the kinetics of the rate-determining Volmer step. Techno-economic analysis indicates a levelized hydrogen production cost of $2.02 kg⁻^1^, meeting the US DOE targets. Our strategy presents a viable pathway to designing next-generation catalysts for industrial AEMWE for green hydrogen production.

**Supplementary Information:**

The online version contains supplementary material available at 10.1007/s40820-025-01798-x.

## Highlights

- A strongly coupled platinum/molybdenum nitrides nanocluster heterostructure has been prepared by using Pt-containing Anderson-type polyoxometalates as precursors.
- The pronounced electronic coupling at the Pt/Mo_2_N cluster interface facilitates the catalytic decomposition of H_2_O through synergistic stabilization of Pt-H^*^ and Mo-OH^*^.
- The optimized Pt/Mo_2_N-NrGO exhibits a remarkably low overpotential, high mass activity, and exceptional long-term durability (>500 h at 1500 mA cm^-2^) in a practical anion-exchange membrane water electrolyzer.

**Supplementary Information:**

The online version contains supplementary material available at 10.1007/s40820-025-01798-x.

## Graphical abstract

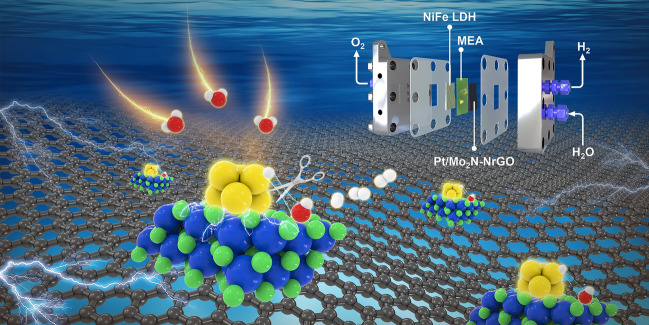

## Introduction

Producing green hydrogen through electrochemical water electrolysis powered by renewable energy sources is regarded as one of the most promising methods for lowering carbon dioxide emission and addressing climate change [1–3]. The anion-exchange membrane water electrolyzer (AEMWE) is a cutting-edge alkaline water electrolysis technology that boasts impressive benefits including high efficiency, compact size, high current density, and rapid response to changes in power, making it ideal for producing green hydrogen [4, 5]. Hitherto, platinum (Pt) has been recognized as the top electrocatalyst for the hydrogen evolution reaction (HER) with low overpotential and long lifespan [6, 7]. However, the HER kinetics of Pt in alkaline conditions is around two orders of magnitude lower than that in acidic environments, primarily due to the sluggish water dissociation and inefficient proton supply [8, 9]. Moreover, the naked Pt electrocatalyst can continually reconstruct through Oswald ripening during the alkaline HER, which can significantly impact its activity, durability and lifetime. [8] In light of this, it is essential to develop rational strategies to optimize the active sites and local chemical environment of Pt catalyst. This would enhance water dissociation, increase proton supply, and prevent surface reconstruction or ripening, all crucial for constructing high-performance AEMWE devices [8, 10, 11].

Engineering heterostructure interfaces help adjust the electron structures at the interface and foster a synergistic effect between the active metal sites and their supports, which is essential for enhancing catalytic performance [12–16]. Recently, various transition metal supports, especially transition metal hydroxides, have been utilized alongside Pt catalysts as co-catalysts to aid in breaking of H–OH bonds and to boost alkaline HER kinetics [8, 17–20]. Nevertheless, this combination may end up obscuring some of active Pt sites on the surface, often leading in a 30–50% reduction in the electrochemical active surface area (ECSA) when compared to those do not include the combination [10, 21]. Therefore, reducing the size of both Pt and the co-catalytic supports could open up more opportunities for the advanced high-performance electrocatalysts. This approach would help avoid the unwanted blockage of surface Pt active sites and enhance the functionality of the co-catalysts [22].

**Fig. 1:**
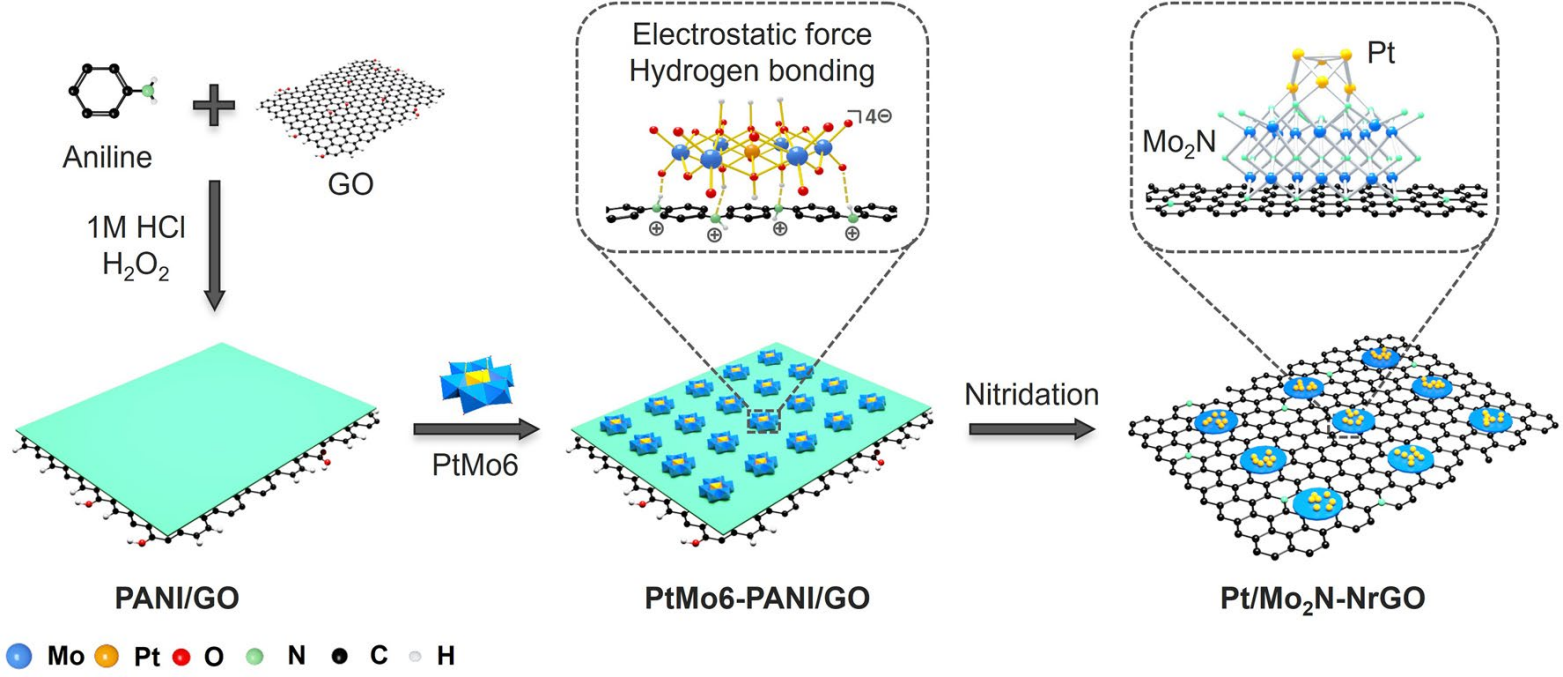
Schematic illustration of synthetic route to the Pt/Mo_2_N-NrGO electrocatalyst

Polyoxometalates (POMs) are inorganic anion clusters with well-defined nanostructures, measuring just 1 ~ 2 nm in size. These characteristics make them excellent molecular platforms for the development of highly efficient electrocatalysts [23–25]. Our previous works have shown that Anderson-type POMs clusters ([XMo_6_O_24_H_6_]^n−^, denoted as XMo6, X represents a transition metal), composed of one heteroatom XO_6_ octahedron with six edge-sharing MoO_6_ octahedrons, can be used to fine-tune the electronic structure of electrocatalysts with precise atomic doping [26]. Moreover, POMs-derived molybdenum nitride (Mo_2_N, a catalyst with high conductivity and strong chemical stability) [27] quantum dots can efficiently lower the energy barriers of water dissociation, which is the key rate-limiting Volmer step for the alkaline HER [28]. Inspired by the above works, we suggest creating a highly coupled cluster heterostructure catalyst to accelerate the sluggish alkaline HER to achieve high-performance AEMWE. The cluster heterostructure features Pt and Mo_2_N clusters situated on nitrogen-doped reduced graphene oxide (denoted as Pt/Mo_2_N-NrGO). It is developed using a Pt-containing Anderson-type POMs cluster (demoted as PtMo6), as the precursor (Fig. 1). Importantly, the well-defined PtMo6 POMs cluster with inherent Pt–O–Mo covalent bonds promote strong interfacial bonding between Pt and Mo_2_N clusters in Pt/Mo_2_N–NrGO. This leads to a wealth of co-catalytic active sites at the interface, significantly enhancing the kinetics of alkaline HER. The resulting co-catalytic effect can be directly observed by operando Raman spectroscopy technique during the alkaline HER process, revealing simultaneous binding of Pt with H and Mo_2_N with OH. Moreover, the Mo-OH* intermediates can serve as a reservoir, continuously supplying protons to Pt active sites. As a result, the Pt/Mo_2_N–NrGO electrocatalyst exhibits excellent alkaline HER performance in both half-cell setup and AEMWE device. This work could inspire further investigations into the rational design of POMs clusters to develop more efficient cluster heterostructures that achieve both high activity and stability in electrocatalysis and other catalysis applications.

## Experimental Section

### Materials

Sodium hexahydroxyplatinate (IV) (Na_2_Pt(OH)_6_, AR 99.9%) was obtained from Shanghai Haohong Scientific Co., Ltd. Potassium chloroplatinate (K_2_PtCl_6_, RG 99.95%) was purchased from Adamas-beta®. Ammonium molybdate tetrahydrate (H_24_Mo_7_N_6_O_24_·4H_2_O, AR 99%), nitric acid (AR) and platinum on carbon (20% Pt/C) were purchased from Shanghai Macklin Biochemical Co., Ltd. Aniline (C_6_H_7_N, ACS ≥ 99.5%) was purchased from Energy Chemical. Hydrochloric acid (AR), isopropyl alcohol (HPLC ≥ 99.7%) and hydrogen peroxide aqueous solution (30% H_2_O_2_, AR) were obtained from Sinopharm Chemical Reagent co., Ltd. Urea (AR) was purchased from Aladdin®. Nafion perfluorinated resin solution was purchased from Shanghai Hesen Electric Co., Ltd. Carbon paper and anion-exchange membrane were obtained from SCI Materials Hub. All above reagents were used without further purification unless otherwise stated.

### Characterizations

Fourier transform infrared spectroscopy was recorded on SHIMADZU IRTracer-100 FE-IR spectrometer. X-ray diffraction characterization was measured by PANalytical B.V. X-ray diffractometer using Cu-Kα radiation. ICP-AES was performed by Agilent 720ES inductively coupled plasma emission spectrometer. Scanning electron microscopy (SEM) images were undertaken using an FEI NovananoSEM 450. Transmission electron microscopy (TEM) images were conducted on an Thermo fisher spectra 300 filed emission spherical aberration correction transmission electron microscopy. Energy dispersive X-ray (EDS) was performed on Super X spectrometer. Holey carbon-formvar support films were purchased by Zhongjingkeyi (Beijing) Film Technology Co., Ltd. The silicon nitride membrane was purchased from YW MEMS. X-ray photoelectron spectroscopy (XPS) was measured on a Thermo fisher Escalab 250Xi with Al-Kα radiation calibrated with C 1*s* (284.8 eV). Raman spectra was carried out on Renishaw Qontor confocal Raman microscope operating at 532 nm using Ar-ion laser. Tube furnace was performed from Hefei Kejing Materials Technology Co., Ltd. Electrochemical workstation was measured on CHI760E purchased by Shanghai CH Instruments.

### Preparation of PtMo6, Pt/Mo_2_N–NrGO, Pt–NrGO, Mo_2_N–NrGO, and NrGO

#### Preparations of the PtMo6

The PtMo6 (Na_4_[H_4_PtMo_6_O_24_]·2H_2_O) precursor was synthesized according to the reported literature [29]. Firstly, 137 mg Na_2_Pt(OH)_6_ was dissolved in 20-mL deionized (DI) water with vigorous stirring. Secondly, 494.2 mg H_24_Mo_7_N_6_O_24_·4H_2_O was dissolved in 30-mL DI water by magnetic stirring. Then, the Na_2_Pt(OH)_6_ solution and H_24_Mo_7_N_6_O_24_·4H_2_O solution were mixed together, and the pH value was kept at about 5.4 by adding 3 M HNO_3_ dropwise. Subsequently, the above mixture was heated to 80 °C and continuously evaporated to 20 mL. After cooling, the pale-yellow crystals of PtMo6 were isolated and dried at room temperature.

#### Preparations of the Pt/Mo_2_N–NrGO

The PANI/GO suspension was acquired from our previous work [28]. In short, aniline (100 mg) that was dissolved in 10 mL HCl solution was mixed with 20-mL graphene oxide (GO) (0.25 mg mL^−1^) solution with vigorous stirring. After 30 min, 500 μL H_2_O_2_ solution was added into the above solution and persistently stirred for 24 h. Then, the bottle-green suspension of PANI/GO was obtained after centrifugation and washing by DI water. Subsequently, 2 mg PtMo6 that dissolved into 15 mL DI water mixed with 20 mL PANI/GO solution with stirring. Then, the PtMo6-PANI/GO powder was obtained by the freeze-drying. Eventually, one equivalent of PtMo6-PANI/GO and two equivalents of urea were ground uniformed and calcined in a tube furnace at 800 °C in H_2_/Ar atmosphere for 3 h to produce Pt/Mo_2_N-NrGO.

#### Preparations of the Pt–NrGO, Mo_2_N–NrGO, and NrGO

The controlled Pt-NrGO, Mo_2_N-NrGO and NrGO samples were also prepared by the similar method to Pt/Mo_2_N-NrGO. The Pt-NrGO was prepared without the addition of H_24_Mo_7_N_6_O_24_·4H_2_O, while the Mo_2_N-NrGO was prepared without the addition of K_2_PtCl_6_. The NrGO was obtained from directly calcining PANI-GO without using K_2_PtCl_6_ and H_24_Mo_7_N_6_O_24_·4H_2_O precursors.

### Electrochemical Measurements

The HER performances were tested by standard three-electrode configuration in 1.0 M KOH at room temperature. Firstly, 5 mg catalysts were dispersed into the mixture of 900 μL isopropyl alcohol and 100 μL Nafion resin (5 wt%) to get a uniform ink. The working electrode was obtained by coating above catalysts ink on carbon paper with an area of 1 cm^2^ and a loading mass of 0.5 mg cm^−2^. According to the ICP-AES results, the Pt/Mo_2_N–NrGO catalyst contained 14.932% Mo and 3.394% Pt. As a result, 76.60 μg cm^−2^ of Mo and 16.97 μg cm^−2^ of Pt were loaded on the working electrode surface. The graphite rod and reversible hydrogen electrode (RHE) were used as counter and reference electrodes, respectively. The 20 wt% Pt/C catalyst was measured as comparison. A manual 95% iR compensation was applied to offset the resistance during the experiments. Linear sweep voltammetry (LSV) was tested from 0.0 to − 0.5 V (the voltages were relative RHE electrode if not otherwise indicated in this paper) at a scan rate of 5 mV s^−1^ and the overpotential (*η*) can be read from LSV curves. Cyclic voltammetry (CV) was conducted for assessing Cdl from 0.1 to 0.2 V with sweep rates of 20, 40, 60, 80, and 100 mV s^−1^, respectively. Electrochemical impedance spectroscopy (EIS) was probed at various overpotentials by applying an AC amplitude of 5 mV within 0.1 to 10,000 Hz frequency range.

Mass activity (MA) can reflect the intrinsic catalytic activity of catalysts. It was evaluated by normalizing the LSV current with catalytic active site weight, which is calculated by Eq. 1:

1 $$\text{MA}=\frac{j}{m}$$

where *j* was read from LSV polarization current and *m* was calculated by ICP-AES measurements.

Turnover frequency (TOF) was also an important parameter that can determine the catalytic activity at a certain overpotential, which is calculated according to Eq. 2:

2 $$\text{TOF}=\frac{j\times S}{2\times F\times n}$$

where *j* was the current density from LSV curves. *S* represented the geometric surface area of electrocatalysts. *F* was faraday constant (96,485 C mol^−1^), and *n* was the mole amount of active metal atoms calculated by the ICP-AES measurements.

Faraday efficiency (FE) represented the ability to produce hydrogen gas from electrons transfer based on Eq. 3:

3 $$\text{FE}=\frac{Q\times V}{2\times F}$$

where Q was calculated by Q = ${\int }_{0}^{t}idt$ from *i-t* plot. V was the gas molar volume at 25 °C and 1.01 × 10^5^ Pa.

### Anion-Exchange Membrane Water Electrolyzer Testing

The AEMWE device with serpentine flow channel, effective area of 2 × 2 cm^2^, was used to test practical applications of Pt/Mo_2_N–NrGO electrocatalyst. This system consisted of a cathode with Pt/Mo_2_N–NrGO on carbon paper, an anode with NiFe-layered double hydroxide (NiFe LDH) and an anion-exchange used Piperion AEM-a40 anion-exchange membrane (activated by soaking in a 1.0 M KOH solution). Pt/Mo_2_N–NrGO or commercial 20 wt% Pt/C electrocatalysts were dispersed into mixture solution include ethanol and Piperion AEM-a40 polymer ionomer, which were then sprayed onto one side of the anion-exchange membrane. The loading mass of Pt/Mo_2_N–NrGO or commercial 20 wt% Pt/C was set to 1.5 mg cm^−2^. The AEMWE system was operated in 1.0 M KOH at 80 °C with peristaltic pump at flow rate of 60 mL min^−1^. The polarization curves were obtained from 1.3 to 2.0 V. The long-term stability tests were performed at a current density of 1.5 A cm^−2^. The energy efficiency of this AEMWE system is calculated as below:

4 $$\text{Energy efficiency}=\frac{E\text{output}}{E\text{input}}=\frac{M(H2) \times HHV}{E\text{input}}$$

where the E_input_ was the electric power consumed to produce the hydrogen-based HHV condition, which was used in previous literatures. The E_output_ was the energy released through the combustion of all generated hydrogen. M_H2_ represents the weight of hydrogen gas production. HHV was the highest value of H_2_ (141.7 kJ g^−1^).

### XAS Measurements and Analysis

X-ray absorption fine structure (XAFS) spectra at Pt L_3_-edge was measured on the 1W1B beamline of Beijing Synchrotron Radiation Facility (BSRF) operated at 2.5 GeV and 250 mA. The data and the corresponding reference samples were measured in transmission mode. Athena software package was used to normalize the data of EXAFS profiles. The Fourier transform (FT) data of Pt L_3_-edge spectra were analyzed from the structure model of Pt/Mo_2_N–NrGO for the Pt–N, Pt–Mo and Pt–Pt scattering paths.

### *In Situ* Electrochemical Raman Measurements

The conductive copper foil was washed with DI water and ethanol by sonicating during 30 min. Then, the 100 μL Pt/Mo_2_N-NrGO catalysts ink was drop-cast onto the surface of the copper foil and dried naturally to be working electrode. Meanwhile, the Pt wire and Hg/HgO electrode were used as counter electrode and reference electrode, respectively. The operando Raman spectroscopic experiments were performed in a Renishaw Qontor confocal Raman microscope system. The Raman wavelength of the semiconductor laser was 532 nm using Ar-ion laser and the Raman frequencies were calibrated using silicon wafers. An CHI760E electrochemical workstation was used to control the potentials.

### Density Functional Theory (DFT) Calculations

The spin-polarized first-principles calculations were performed using the periodic DFT method implemented in the DMol3 software package [30]. The exchange–correlation energy was determined using the generalized gradient approximation, specifically employing the Perdew–Burke–Ernzerhof (PBE) functional. The Grimme’s PBE + D2 method was employed to account for the long-range dispersion interaction. For core treatment, a density functional semicore pseudopotential method was utilized, while the valence electrons were described using a double-numerical basis with polarization functions (DNP). A (8 × 8 × 1) k-point grid was employed to sample the Brillouin zone. The transition states were explored using the complete linear synchronous transit/quadratic synchronous transit method and subsequently validated through frequency calculations. Convergence tolerances were set at 1 × 10^–5^ Ha, 2 × 10^–3^ Ha Å^−1^, and 5 × 10^–3^ Å for energy, gradient and displacement, respectively. Pt/Mo_2_N was constructed using optimized six Pt atoms on Mo_2_N(111) surface with (4 × 4) unit cell, with the bottom two layers of atoms fixed and the remaining atoms fully relaxed with a vacuum layer of 20 Å built on top of the Pt/Mo_2_N. The Mo_2_N(111) surface was modeled using a (4 × 4) unit cell with a four-layer slab and a 20 Å vacuum region. The atoms in the two bottom layers were fixed, while the other atoms underwent full relaxation. The Pt(111) surface was modeled using a (4 × 4) unit cell with a four-layer slab and a 20 Å vacuum region. The atoms in the bottom two layers were constrained, while the remaining atoms underwent full relaxation. The change of free energy (ΔG) of reaction intermediates was calculated using the following equation:

7 $$\Delta {\text{G}} = \Delta {\text{E}} + \Delta {\text{ZPE}}{-}{\text{T}}\Delta {\text{S}}$$

where ΔE was the change of electronic energy between the reactant and product based on static electronic self-consistent calculations, ΔZPE was the change of the zero-point energy, and ΔS represented the change in entropy at temperature T = 298 K, respectively.

The d-band center (${\varepsilon }_{\text{d}}$) was calculated by:

8 $${\varepsilon }_{\text{d}}=\frac{{\int }_{-\infty }^{+\infty }E{\rho }_{\text{d}}\left(E\right)\text{d}E}{{\int }_{-\infty }^{+\infty }{\rho }_{\text{d}}\left(E\right)\text{d}E}$$

where ${\varepsilon }_{\text{d}}\left(E\right)$ are the projected electron density of d state at the level of *E*.

## Results and Discussion

### Synthesis and Characterizations of Pt/Mo_2_N–NrGO

The overall synthetic route of Pt/Mo_2_N-NrGO cluster heterostructure electrocatalyst is illustrated in Fig. 1 (details see Experimental Section). First, protonated polyaniline coated graphene oxide (PANI/GO) is obtained by the in situ oxidative polymerization of aniline on the GO surface according to our previous works [28]. The PtMo6 Anderson-type POMs anion clusters [29, 31] (Fig. S1) are anchored on positively charged PANI/GO substrate through electrostatic force and hydrogen bonding, after nitridation, creating the PtMo6-PANI/GO nanosheets. SEM, TEM and high-angle annular dark-field scanning transmission electron microscopy (HAADF-STEM) reveal that the PtMo6 POMs clusters are well dispersed in PtMo6-PANI/GO nanosheets (Figs. S2 and S3). Moreover, the characteristic infrared absorption peaks of Mo = O (933 cm^−1^), Mo–O–Mo (661 cm^−1^), Pt–O–Mo (518 cm^−1^), and Pt–O (424 cm^−1^) of PtMo6 cluster distinctly appear in PtMo6–PANI/GO, demonstrating the successful formation of PtMo6 POMs cluster and PANI/GO substrate interface (Fig. S2d) [29]. The catalyst featuring a strongly coupled cluster heterostructure, Pt/Mo_2_N-NrGO, is synthesized by the nitridation of PtMo6-PANI/GO precursor. After nitridation, the porous nanosheet structure of Pt/Mo_2_N-NrGO is retained (Fig. S4).

**Fig. 2:**
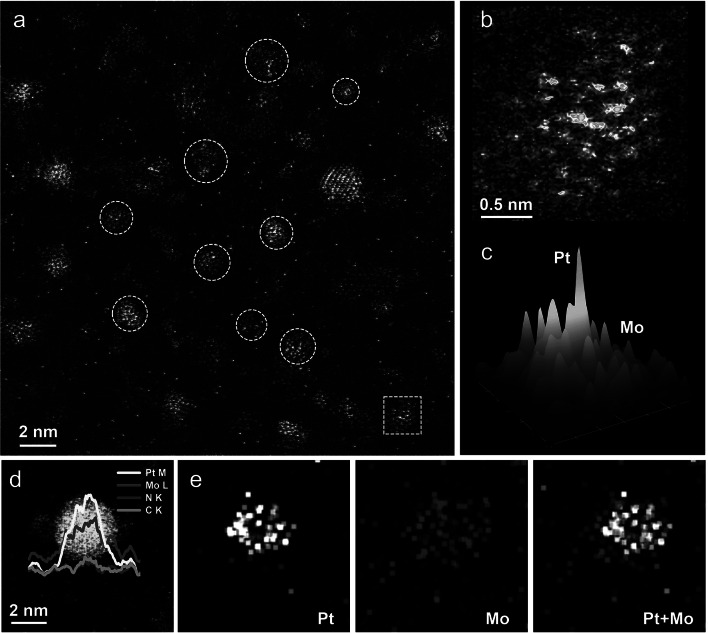
Morphology characterizations of Pt/Mo_2_N-NrGO catalyst. **a** Aberration-corrected HAADF-STEM image, red circles represent the Pt/Mo_2_N clusters. **b** 2D intensity range profile shown for the red dashed square in image (**a**). **c** 3D surface intensity plot of image (**b**). **d** Magnified AC HAADF-STEM image of single nanocluster with linear intensity profiles. **e** The corresponding STEM-EDS element mappings of Pt/Mo_2_N–NrGO cluster in image (**d**)

The HADDF-STEM image shows that the majority of Pt/Mo_2_N nanoclusters, averaging 2.0 nm in diameter, are uniformly embedded on the Pt/Mo_2_N–NrGO nanosheet (Fig. S4). The aberration-corrected HAADF-STEM has also been carried out to probe the distribution state of Pt and Mo_2_N clusters. In addition to a small amount of Pt single atoms loaded on the Mo_2_N clusters, most of Pt showed intermetallic nanoclusters with Mo_2_N clusters (Fig. 2a). Moreover, the two-dimensional (Fig. 2b) and three-dimensional (Fig. 2c) intensity surface maps of dashed square clusters in Fig. 2a can intuitively distinguish the Pt atoms and Mo_2_N clusters based on the difference of Z-contrast intensity, which confirms the strong interfacial interaction between Pt and Mo_2_N in Pt/Mo_2_N–NrGO. The bright and dark contrast between Pt and Mo is due to their different Z-contrast, which is proportional to the 1.7th power of the atomic number (Z) [32]. The intensity ratio of Pt to Mo is 3.04, which is close to the theoretical ratio of 2.80 (Z_Pt_^1.7^: Z_Mo_^1.7^) obtained by simulating the crystal structure (Fig. S5). As a control, the Mo_2_N-loaded NrGO (Mo_2_N–NrGO) and Pt-loaded NrGO (Pt–NrGO) have also been synthesized using the same method without the addition of Pt and Mo precursors, respectively (Figs. S6 and S7). Both of Mo_2_N–NrGO and Pt–NrGO composites exhibit severe agglomeration, showing average cluster sizes being 3 ~ 6 times larger than that of Pt/Mo_2_N–NrGO. The lattice fringes with d-spacing of 0.25 nm can be assigned to the (111) crystal plane of Mo_2_N (PDF#25–3166), whereas the brighter counterpart can be deduced to be Pt clusters (Fig. S8).

In addition, the STEM-coupled energy-dispersive spectroscopy (EDS) element mappings corroborate that the Pt and Mo species are concomitantly connected on the NrGO (Fig. S9). The STEM-EDS line intensity analysis is further performed to confirm the presence of Pt/Mo_2_N cluster heterostructure in the Pt/Mo_2_N-NrGO catalyst (Fig. 2d). The corresponding atomically resolved elemental mappings show that the Pt is concentrated in the center of the Mo element (Fig. 2e), indicating the strongly coupled interface between Pt and Mo_2_N clusters. The contents of Pt and Mo in Pt/Mo_2_N–NrGO catalysts are determined to be 3.39 and 14.92%, respectively, by the inductively coupled plasma-atomic emission spectrometry (ICP-AES) and the atomic ratio of Mo to Pt is about 4.45, which is close to that of PtMo6 POMs precursor (Table S1). It should be noted that the atomic contents of Mo and Pt derived from the EDS scan line profile in Fig. 2d is different from the average element contents of Mo and Pt from the ICP-AES results. This is reasonable since the PtMo6 precursors show higher atomic content of Mo than Pt, but if the Pt atoms accumulate to Pt clusters on the Mo_2_N clusters, the Pt may possess a higher content relative to Mo. The above results demonstrate that the strongly coupled Pt/Mo_2_N cluster heterostructure catalyst is successfully facilitated by using the well-defined PtMo6 POMs cluster as a precursor, which may play a crucial role in improving the alkaline HER activity and durability of Pt/Mo_2_N-NrGO electrocatalyst.

**Fig. 3:**
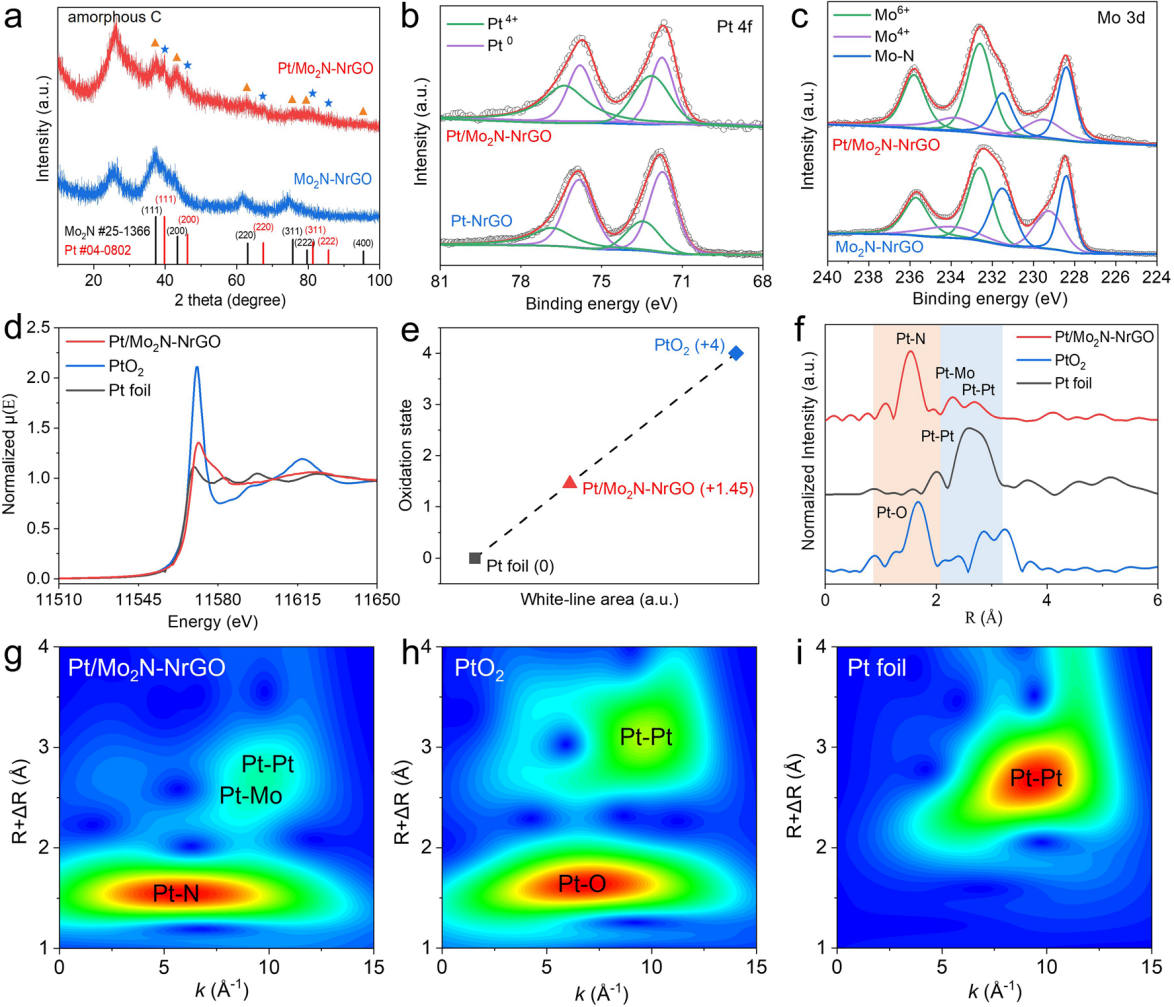
Structure characterizations of Pt/Mo_2_N–NrGO catalyst. **a** XRD patterns of Pt/Mo_2_N-NrGO and Mo_2_N-NrGO. High-resolution XPS spectra of **b**. Pt 4*f* and **c** Mo 3*d*. **d** Normalized XANES of Pt L_3_-edge of Pt/Mo_2_N-NrGO, PtO_2_ and Pt foil. **e** The fitted average oxidation states of Pt from XANES spectra. **f** EXAFS spectra of Pt/Mo_2_N-NrGO, PtO_2_ and Pt foil. Wavelet transform images of **g** Pt/Mo_2_N-NrGO, **h** PtO_2_, and **i** Pt foil

The characteristic peaks in XRD patterns of Pt/Mo_2_N-NrGO are located at 37.30°, 42.76°, 62.55°, and 75.73°, which can be well indexed to the corresponding to the (111), (200), (220), and (311) crystal planes of the cubic Mo_2_N (PDF#73–1768) [33], respectively (Fig. 3a). In addition, the weak peaks located at 39.75°, 46.16°, 67.31°, and 79.23° are attributable to (111), (200), (220), and (311) crystal planes of Pt (#04–0802). The main peak of Pt/Mo_2_N-NrGO is located at 37.30°, which has undergone a significant left shift compared to that of Mo_2_N–NrGO (35.57°), indicating that the increase in lattice spacing of Mo_2_N (tensile strain) after introducing Pt. Compared with the XRD of Pt-NrGO (Fig. S10), Pt/Mo_2_N–NrGO only contains a much lower signal of Pt, indicating that the Mo_2_N support is able to significantly inhibit the agglomeration of Pt atoms (Table S2). Furthermore, the peaks of Mo_2_N in Pt/Mo_2_N–NrGO are broader and lower than those in Mo_2_N–NrGO, indicating that the Pt atom in PtMo6 POMs clusters can effectively prevent the agglomeration of Mo_2_N species. The full width at half maximum (FWHM), lattice parameters and crystallite sizes of Pt and Mo were provided in Table S2. The particle sizes of Pt/Mo_2_N–NrGO and Mo_2_N–NrGO were estimated from the FWHM values (Table S2), consistent to the HRTEM results. The presence of graphene carbon matrix in Pt/Mo_2_N–NrGO can be verified by the Raman spectra (Fig. S11). Nitrogen adsorption measurement of Pt/Mo_2_N–NrGO provides a Brunauer–Emmett–Teller (BET) specific surface area (SSA) of 94.56 m^2^ g^−1^ and an average pore size of 2.34 nm (Fig. S12). XPS studies verify the existence of Pt, Mo, C, N, and O elements in the synthesized Pt/Mo_2_N–NrGO catalyst (Fig. S13). As shown in Fig. 3b, the Pt in Pt/Mo_2_N–NrGO exists in two different oxidation phases. Two characteristic peaks for Pt^0^ were located at 72.05 eV (Pt^0^ 4*f*_7/2_) and 75.35 eV (Pt^0^ 4*f*_5/2_), respectively, while those for Pt^4+^ were located at 72.52 eV (Pt^4+^ 4*f*_7/2_) and 75.90 eV (Pt^4+^ 4*f*_5/2_), respectively [34, 35]. The integral area ratio of Pt^4+^: Pt^0^ in Pt/Mo_2_N-NrGO catalyst shows a striking increase from 0.50 to 1.16, compared to that of Pt-NrGO, indicating the Pt species on the Mo_2_N clusters tend to be oxidized more easily. Besides, for high-resolution Mo 3*d* (Fig. 3c and Table S3), the peaks at 228.48 and 231.48 eV can be ascribed to Mo 3*d*_5/2_ and Mo 3*d*_3/2_ of Mo–N species, respectively. [33] The binding energy of 229.48 and 233.78 eV can be ascribed to Mo 3*d*_5/2_ and Mo 3*d*_3/2_ of Mo^4+^ in MoO_2_, respectively. The peaks located at 232.58 and 235.78 eV can be attributed to Mo 3d_5/2_ and Mo 3*d*_3/2_ of Mo^6+^ in MoO_3_, respectively [36, 37]. Interestingly, the ratio of MoO_x_ to Mo_2_N decreases from 0.65 (Mo_2_N–NrGO) to 0.59 (Pt/Mo_2_N–NrGO), further confirming the electrons transfer from Pt to the Mo_2_N cluster due to the strongly coupled Pt/Mo_2_N interface (Table S3). The loss of electrons from the Pt clusters improves the adsorption energy between Pt and the active H*, which is conducive to lowering the activation energy of the Tafel process and accelerate the release of H_2_ [38, 39]. The X-ray absorption near-edge spectroscopy (XANES) of the Pt L3-edge in Fig. 3d illustrates that the white-line intensity of Pt in Pt/Mo_2_N-NrGO is between PtO_2_ (Pt^4+^) and Pt foil (Pt^0^). The average oxidation state of Pt (+ 1.45) in Pt/Mo_2_N–NrGO catalyst is higher than 0 in Pt foil, representing the electrons are partially transferred from Pt to the Mo_2_N cluster, which is consistent with the XPS results (Fig. 3e, b, c) [34, 40]. The Fourier transform extended X-ray absorption fin structure (FT-EXAFS) was further analyzed to confirm that the dominant Pt–N–Mo bonding at the interface of Pt and Mo_2_N clusters in the as-prepared Pt/Mo_2_N–NrGO (Fig. 3f). The EXAFS signal of Pt/Mo_2_N–NrGO exhibits a major peak of Pt–N at 1.56 Å, shorter than Pt-O bond of PtO_2_ (1.66 Å), indicating Pt–N bonds probably bridge the Pt and Mo atoms in the interface between Pt and Mo_2_N clusters. The Pt–N coordination bond in this first shell layer originates from the Pt–N-Mo bond, the dominant bonding mode in Pt/Mo_2_N-NrGO, bridging the Pt cluster to the Mo_2_N substrate. The peak located at 2.30 Å in the second coordination layer can be attributed to the Pt–Mo bond. The second shell layer of Pt/Mo_2_N-NrGO has a weak satellite peak at 2.69 Å, which is close to the position of the Pt–Pt bonding peak (2.63 Å) in the Pt foil and is attributed to the bonding of Pt clusters in Pt/Mo_2_N-NrGO. The Pt–N, Pt–Mo, and Pt–Pt contributions probably originate from the interaction between Pt cluster and over Mo_2_N (111) facets. To visually distinguish the Pt–N, Pt–Mo, and Pt–Pt bonds, the wavelet transform (WT) of Pt L3-edge oscillations representation of the EXAFS signal for Pt foil, Pt/Mo_2_N-NrGO and PtO_2_ are provided (Fig. 3g-i) [41]. The Pt/Mo_2_N–NrGO sample exhibits the primary signal of Pt–N coordination in the *k*-space, which is located at 5.69 Å^−1^, which is different from that of Pt-O bond (6.83 Å^−1^) in PtO_2_. Moreover, the Pt–Mo bond of Pt/Mo_2_N-NrGO in the k-space is located at 8.59 Å^−1^, lower than those of Pt–Pt bonds in PtO_2_ (9.47 Å^−1^) and Pt foil (9.52 Å^−1^), which can be well distinguished from Pt–Pt bonding in PtO_2_ and Pt foil (Fig. 3g–i). The above XANES and EXAFS analysis not only confirm the charge transfer between Pt and Mo_2_N clusters but also suggest the dominant Pt–N-Mo bonding at the interface of Pt and Mo_2_N clusters in the as-prepared Pt/Mo_2_N-NrGO electrocatalyst.

### Electrochemical HER Performance of Pt/Mo_2_N–NrGO

**Fig. 4:**
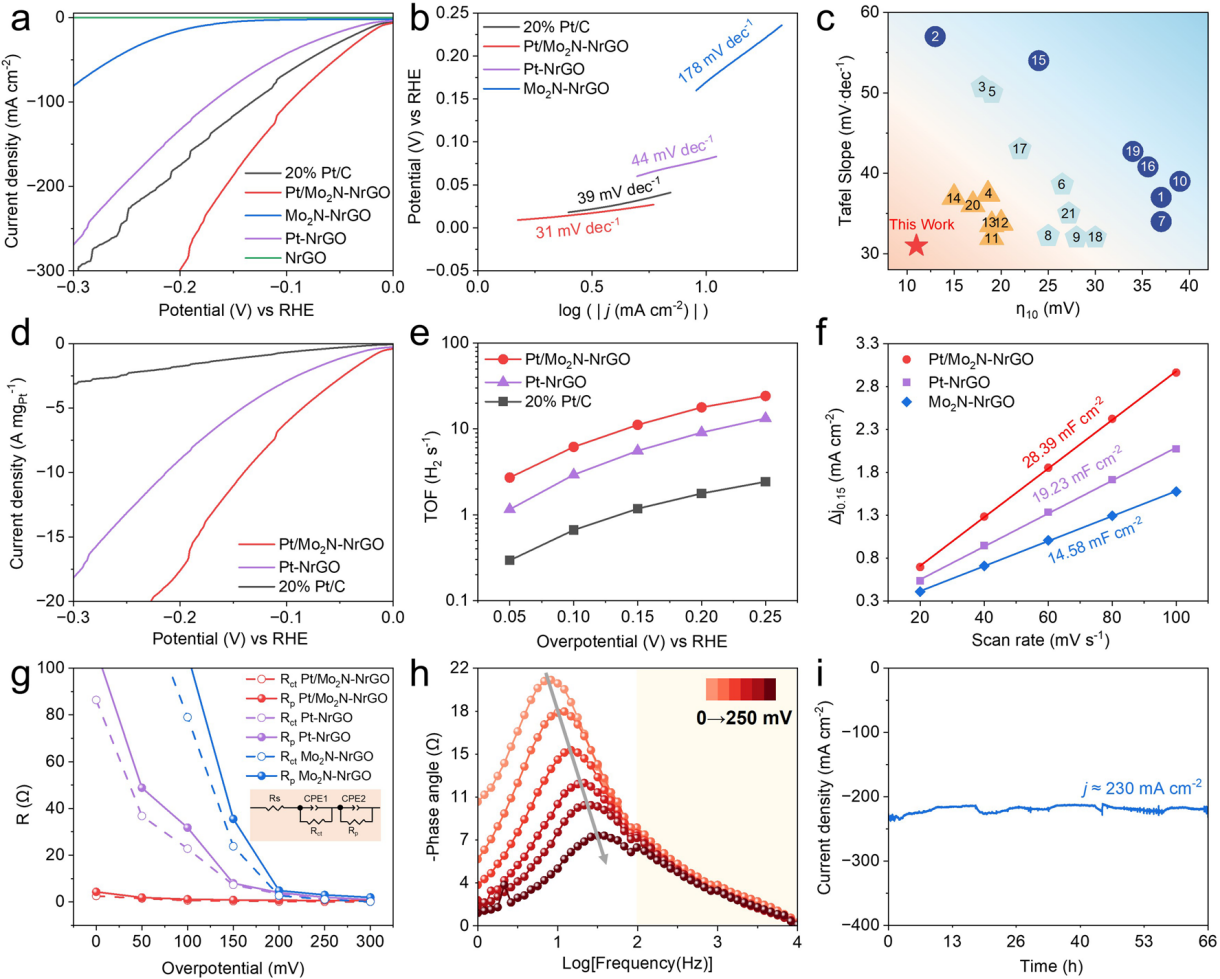
HER performance. **a** LSV curves of 20% Pt/C, Pt/Mo_2_N-NrGO, Pt-NrGO, Mo_2_N-NrGO, and NrGO.** b** Tafel slope diagrams of 20% Pt/C, Pt/Mo_2_N-NrGO, Pt-NrGO, and Mo_2_N-NrGO. **c** Comparison of merits concerning Tafel slope and overpotential at η_10_ plotted from Table S4. **d** Mass activity and **e** turnover frequency of 20% Pt/C, Pt/Mo_2_N-NrGO, and Pt-NrGO. **f** Double-layer capacitance comparison of Pt/Mo_2_N-NrGO, Pt-NrGO, and Mo_2_N-NrGO. **g** Plots of simulated resistance values of Rct and Rp (inset: the equivalent circuit model). **h** Bode phase plot for Pt/Mo_2_N-NrGO at different overpotentials from 0 to 250 mV.** i** Chronoamperometry response curve of Pt/Mo_2_N-NrGO at 180 mV versus RHE

The alkaline HER performance of the Pt/Mo_2_N–NrGO electrocatalyst is initially assessed using a standard three-electrode system. The Pt/Mo_2_N–NrGO exhibits an ultralow overpotential of merely 11 mV to reach a current density of 10 mA cm^−2^, surpassing those of Mo_2_N–NrGO, Pt–NrGO, NrGO and commercial Pt/C catalysts (Fig. 4a). Moreover, the Tafel slope of Pt/Mo_2_N–NrGO (31 mV dec^−1^) is much lower than those of Pt-NrGO (44 mV dec^−1^), Mo_2_N–NrGO (178 mV dec^−1^), even commercial Pt/C (39 mV dec^−1^) and those achieved previously with Pt-based catalysts in alkaline conditions, demonstrating the Pt/Mo_2_N-NrGO proceeds via a Volmer-Tafel mechanism similar to that in an acidic environment (Fig. 4b, c and Table S4) [42, 43]. This result manifests that the strongly coupled Pt/Mo_2_N clusters enhance the cleavage of H-OH bond and ensure efficient proton transfer to the Pt active sites, fundamentally altering the acidic-like Tafel step limited kinetics.

Moreover, the Pt/Mo_2_N–NrGO catalyst shows an ultrahigh mass activity (MA) value of 17.72 A mg_Pt_^−1^ at *η*_200_ in 1.0 M KOH (Fig. 4d), which is 10 times higher than that of commercial Pt/C (1.75 A mg_Pt_^−1^). The intrinsic HER activity is also evaluated by calculating the turnover frequency (TOF) value based on the estimated number of Pt active sites. Impressively, the TOF value of Pt/Mo_2_N-NrGO reaches up to 17.82 H_2_ s^−1^ at *η*_200_ (Fig. 4e), which is 10 times higher than that of the 20% Pt/C (1.77 H_2_ s^−1^). We note the TOF value achieved with Pt/Mo_2_N-NrGO is also higher than those of the reported Pt-based catalysts (Table S4). The electrochemical double-layer capacitances (C_dl_), proportional to the electrochemical active surface area (ECSA), is an important parameter to character exposed catalytic active sites via measuring different scan rates of cyclic voltammetry curves (CV) in a non-faraday region (Figs. 4f and S14). The Pt/Mo_2_N–NrGO catalyst exhibits a much higher C_dl_ (28.39 mF cm^−2^) than Pt-NrGO (19.23 mF cm^−2^) and Mo_2_N–NrGO (14.58 mF cm^−2^), suggesting the exposure of much more active sites. EIS at the open-circuit voltage (Fig. S15a) further shows that the arc-radius of Pt/Mo_2_N–NrGO in the Nyquist plot is much smaller than those of Pt–NrGO and Mo_2_N–NrGO, suggesting that lower charge transfer resistance and faster HER kinetics of Pt/Mo_2_N–NrGO. The Nyquist plots measured by operando EIS are simulated using an equivalent circuit model to analyze the hydrogen adsorption kinetics at different potentials (Fig. S15b–d). The simplified equivalent circuit plot (inset in Fig. 4g) depicts R*s*, R*ct*, and R*p*, reflecting the solution resistance, charge transfer resistance, and H* absorption resistance during the HER process, respectively. The quantitative values fitted for Nyquist plots at various overpotentials are summarized in Table S4. The Pt/Mo_2_N–NrGO catalyst shows the lowest values of the Rct and Rp with plateau-like patterns in comparison to a sharp decline in R_ct_ and R_p_ values of Mo_2_N–NrGO and Pt–NrGO (Fig. 4g), suggesting such a strongly coupled Pt/Mo_2_N cluster heterostructure helps to enhance the H* adsorption and accelerate the transfer of intermediate H* species [44]. Meanwhile, the electron conduction rate is characterized by the Bode plots from the response of the phase angle. As shown in Fig. 4h, the Bode plot of Pt/Mo_2_N–NrGO is distinguished into two parts of low-frequency (white) and high-frequency (yellow) regions, which exhibit a smooth phase relaxation, in contrast to the steep decline observed in the Pt–NrGO sample and Mo_2_N–NrGO (Fig. S16), indicating the interaction between intermediates and the Pt/Mo_2_N active sites is enhanced [45]. Additionally, the phase angle of the Pt/Mo_2_N–NrGO catalyst rapidly decreases in the high-frequency region, implying the electron transfer at the inner layer or interface is more efficient than that at the catalyst-electrolyte interface [46]. Therefore, the as-obtained strongly coupled Pt/Mo_2_N interface may help to improve the transfer of H* intermediates and electrons during the HER.

Apart from superior activity, the durability in large ampere-level current density is important for practical applications of electrolyzers. Firstly, the stability of the Pt/Mo_2_N–NrGO catalyst is evaluated using cyclic voltammetry (CV) cycle testing (Fig. S17), which shows no obvious decay of HER activity after 5,000 CV cycles. Additionally, negligible decay can be observed at the cathodic current density of 230 mA cm^−2^ (Fig. 4i) for Pt/Mo_2_N–NrGO catalyst during 66 h of chronopotentiometry test in 1.0 M KOH electrolyte. It is important that no significant aggregation and morphology change after the long-term stability, as revealed by the analysis of XRD, TEM, HRTEM, HAADF-STEM, and STEM-EDS mapping (Fig. S18). The Faradaic efficiency (FE) is also measured to be ~ 99.87% (Fig. S19).

**Fig. 5:**
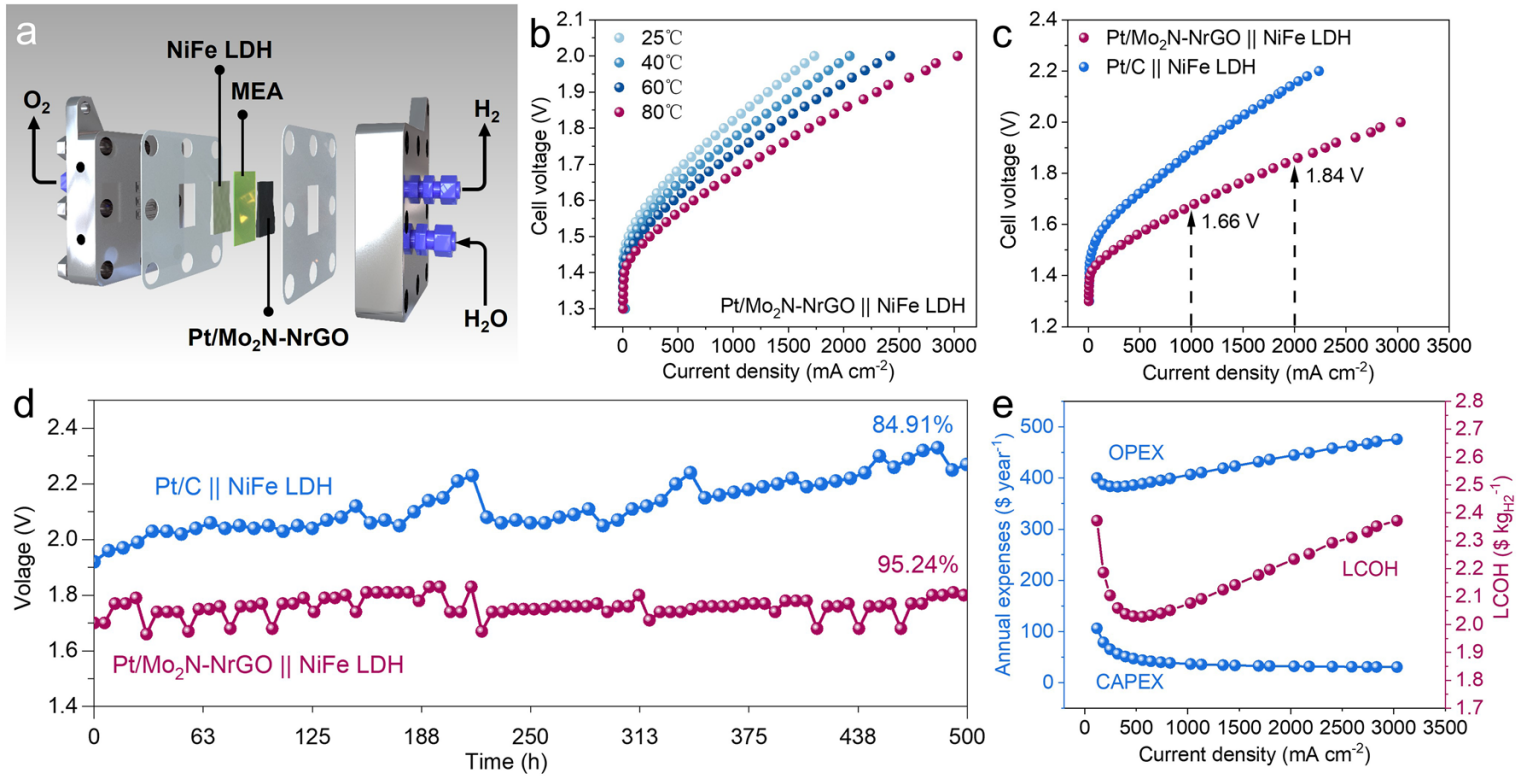
AEMWE performance and techno-economic analysis (TEA) using Pt/Mo_2_N-NrGO as the cathode catalyst. **a** Schematic diagram of the AEMWE in 1.0 M KOH. **b** Pt/Mo_2_N-NrGO || NiFe LDH at various operating temperatures of 25, 40, 60, and 80 °C, respectively. **c** Polarization curves of AEMWEs using Pt/Mo_2_N-NrGO and commercial 20% Pt/C as the cathode catalysts, respectively. **d** Chronopotentiometry curves of AEMWEs (Pt/Mo_2_N-NrGO||NiFe LDH and Pt/C||NiFe LDH) at 1.5 A cm^−2^. **e** Preliminary techno-economic analysis is based on CAPEX, OPEX, and LCOH at different operating current densities

The optimized Pt/Mo_2_N-NrGO is further employed as a cathode catalyst to evaluate the practical application in anion-exchange membrane water electrolyzer (AEMWE) device in 1.0 M KOH electrolyte under 25, 40, 60, and 80 °C, using NiFe-layered double hydroxide (NiFe LDH) and the Piperion AEM-a40 anion-exchange membrane as an anode catalyst and the diaphragm separator, respectively (Figs. 5a, b and S20). For comparison, the commercial 20 wt% Pt/C catalyst loaded carbon paper is also utilized as a cathode for AEMWE. The polarization curves measured for these two aforementioned AEMWE cells indicate that the performance of Pt/Mo_2_N–NrGO || NiFe LDH (AEMWEs are named as cathode || anode) significantly outperforms that of Pt/C || NiFe LDH at 80 °C in Fig. 5c. The industrial-scale current densities of 1.0 and 2.0 A cm^−2^ can be achieved at only 1.66 and 1.84 V, respectively, superior to most of the reported AEMWE catalysts (Table S5). Furthermore, we calculated the H_2_ energy efficiency at a higher heating value (HHV) and TOF values with the variation of current densities (Fig. S21) [47]. The H_2_ energy efficiency of the AEMWE reaches 93.70% and 88.12%, and the TOF values are 50.59 and 92.73 s^−1^ at current densities of 500 and 1000 mA cm^−2^, respectively. Moreover, our chronopotentiometry (CP) studies reveal that the optimized AEMWE device (Pt/Mo_2_N-NrGO||NiFe LDH) exhibits only a slight potential increase (4.76%) at a high current density of 1.5 A cm^−2^ in a 500-h continuous measurement (Fig. 5d), which is the most excellent durability at high current density for AEMWE reported to date (Table S5). The exceptional durability of the AEMWE cell demonstrates the superior structural stability of the Pt/Mo_2_N-NrGO catalyst.

Currently, commercial utilization of hydrogen energy is limited by the high levelized cost of the hydrogen (LCOH). Therefore, a preliminary techno-economic analysis (TEA) has been carried out to evaluate the LCOH based on an ideal 1 MW single AEM cell outlined in this work, assuming the unit price of electricity is $20/MW h. The calculations in this article are derived from an IRENA report titled “Green Hydrogen Cost Reduction” (the details are provided in the supporting information) [48, 49]. Specifically, the TEA includes capital expenditure (CAPEX) and operational expenditure (OPEX). As shown in Fig. 5e, the CAPEX associated with the deployment of the plants rapidly decreases from 106.20 to 31.96 $ year^−1^ as the current density improves from 100 to 2000 mA cm^−2^ in virtue of the diminished number of AEMWE cells to achieve the 1 MW target. On the contrary, the OPEX exhibits a more complex situation contingent upon the diverse electrochemical performance. With the increase in current density, the LCOH mainly depends on the OPEX due to the related flattening of CAPEX. In particular, the actual electric energy consumption progressively takes the largest OPEX shares (supporting information), demonstrating the electrochemical performance of catalysts play a decisive role in decreasing the LCOH. Combining the trends of the CAPEX and OPEX, LCOH reaches the minimum value as low as 2.02 $ kg_H2_^−1^ operated at 560 mA cm^−2^, which is proximity to the US Department of Energy (DOE) metrics of 2 ~ 2.5 $ kg_H2_^−1^, suggesting the Pt/Mo_2_N-NrGO is a promising AEMWE catalyst for large-scale H_2_ production [47].

### Structure–Activity Relationship Investigation

**Fig. 6:**
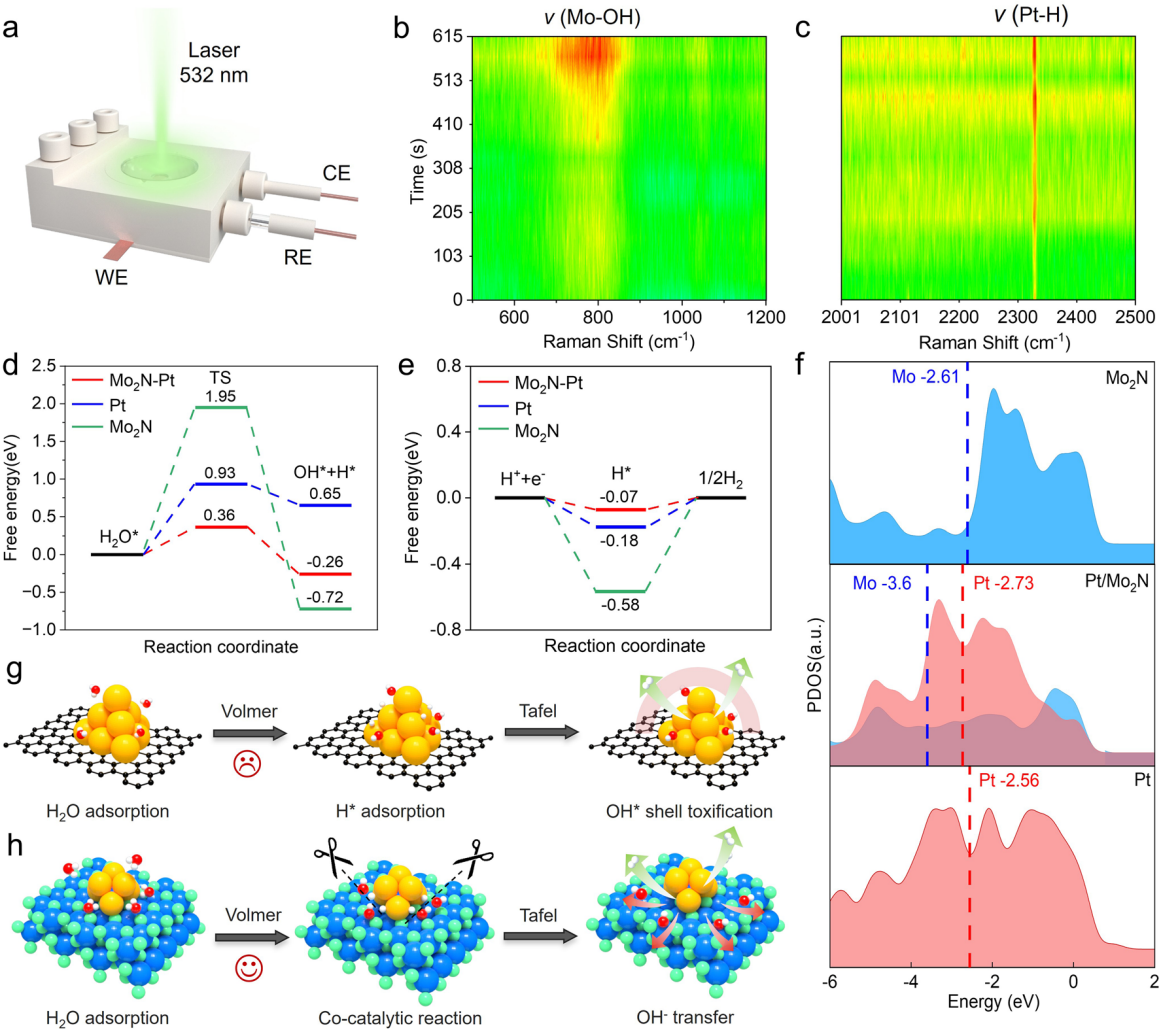
Co-catalytic mechanism of Pt/Mo_2_N-NrGO. **a** Schematic illustration of operando Raman device. **b, c** Operando Raman spectra of Pt/Mo_2_N-NrGO during the alkaline HER. **d** Calculated Gibbs free energy diagram for the Volmer step. **e** Calculated Gibbs free energy diagram for the Tafel step. **f** Density of states (DOS) analysis of Mo 3*d* and Pt 5*d* orbital of Mo_2_N(111), Pt/Mo_2_N-NrGO and Pt(111). Schematic illustration of the mechanism of **g** Pt/C and **h** Pt/Mo_2_N-NrGO catalyst. The orange, blue, red, green, and gray represent Pt, Mo, O, N, H, and C atoms, respectively

To gain deeper insights into the structure–activity relationship, the operando electrochemical Raman spectra are recorded to identify the dynamic evolution of the Pt/Mo_2_N–NrGO catalyst during the alkaline HER process. As shown in Fig. 6a, the operando Raman device comprises a hollow cavity filled with a 1.0 M KOH solution, while a 532 nm Raman laser can be directed irradiated through glass onto the working electrode (WE) surface at the bottom (details see Fig. S22). The signals ranging from 500 to 2500 cm^−1^ are continuously collected at 10 mA·cm^−2^ current density. During the continuous electrocatalytic process, the intensity of peaks at 795.5 (stretching vibrations of Mo–OH skeleton) and 2328.5 cm^−1^ (stretching vibrations of Pt–H terminal bond) sharply increase (Fig. 6b, c) [50–53], indicating H atoms tend to bind with Pt. In contrast, OH groups tend to bind with Mo in the Pt/Mo_2_N cluster heterostructure. The present observation validates the distinctive co-catalytic effect of Pt/Mo_2_N–NrGO catalyst, wherein the kinetics of water dissociation may be accelerated by the strongly coupled interface between Pt and Mo_2_N cluster, and thus significantly enhance the electrocatalytic alkaline HER activity. Interestingly, the intensity of the Pt–H bond runs up and down during the HER, indicating the continuous adsorption of intermediate H* species and subsequent desorption of H_2_ molecules. However, the Mo-OH bond gradually strengthens, suggesting the absorption of OH^−^ on the Mo sites, forming a Mo–OH hydrophilic layer, which may serve as a proton reservoir to continuously deliver protons to Pt active sites, in analogy to natural enzymes with a distinct local chemical environment or channel for the protons transfer. The above operando electrochemical Raman results indicate the co-catalytic effect of the Pt–Mo_2_N interface for enhancing the water dissociation kinetics and accelerating the Volmer step.

Meanwhile, we perform density functional theory (DFT) calculations by taking Pt/Mo_2_N, Pt(111), and Mo_2_N(111) as models to reveal the co-catalytic effect of Pt/Mo_2_N heterostructure (Fig. S23). In a typical alkaline process following the Volmer-Tafel mechanism (confirmed by the experimental results), the H_2_O molecule dissociates on the catalytic sites to generate intermediate H* and OH*, with two neighboring H* subsequently combining to form an H_2_ molecule. Therefore, we calculate the Gibbs energy of the Volmer step and the Tafel step to explain the co-catalytic effect in alkaline media, respectively. As shown in Fig. 6d, the Pt/Mo_2_N has the lowest water dissociation energy barrier (ΔG = 0.36 eV) in comparison with Pt(111) (ΔG = 0.93 eV) and Mo_2_N(111) (ΔG = 1.95 eV), suggesting the rapid HER kinetics for Volmer step. The produced H* species absorbed on the Pt provide an appropriate platform for the continuous generation of H_2_, while the OH* species adsorbed on the Mo site may be beneficial for transferring reactants to the Pt active sites. The calculated adsorption free energy of H* in the Tafel step (ΔG_H*_) on Pt/Mo_2_N (− 0.07 eV) is closer to the thermoneutral value, much smaller than those of Pt(111) (− 0.18 eV) and Mo_2_N(111) (− 0.58 eV) in Figs. 6e and S24–S26. The d-band centers of Pt and Mo in Pt/Mo_2_N, Pt(111) and Mo_2_N(111) are also calculated by using the density of states (DOS) based on d-band theory (Fig. 6f) [52]. In comparison to d-band center of Pt(111) at − 2.56 eV, the left shift of the d-band center of Pt (− 2.73 eV) in Pt/Mo_2_N away from the Fermi level indicates that the Pt has weaker adsorption energy of H*, which is beneficial for the Tafel reaction. Meanwhile, the left shift of the d-band center of Mo (− 3.6 eV) in Pt/Mo_2_N away from the Fermi level compared to that of Mo_2_N(111) at − 2.61 eV, resulting in the weaker adsorption of OH*, which facilitates the migration of OH* on the Mo_2_N surface of Pt/Mo_2_N. Meanwhile, we employed CO stripping voltammetry tests to evaluate the water dissociation ability of Pt/Mo_2_N–NrGO because the OH* can facilitate the removal of adsorbed CO intermediates (Fig. S27) [16]. Compared to the commercial 20 wt% Pt/C (0.69 V) and Pt–NrGO (0.62 V), the desired Pt/Mo_2_N–NrGO catalyst exhibits the lowest peak potential (0.52 V) for CO oxidation, indicating the preference of OH* binding on Pt/Mo_2_N–NrGO catalyst and accelerated kinetics of water dissociation, consistent with the in situ Raman results (Fig. 6b). In this case, the H_2_O molecule can be efficiently dissociated due to the disparate adsorption capacities of H* and OH* on the co-catalytic interface of the Pt/Mo_2_N nanocluster, thereby facilitating the rate-determining Volmer step.

Combining the simultaneously obtained direct evidence of two distinct Pt–H and Mo–OH bonds with the DFT calculation results, we outline a detailed schematic for the novel co-catalytic mechanism of alkaline HER for the Pt/Mo_2_N–NrGO catalyst (Fig. 6g, h). We discuss the dissociation of interfacial water, the adsorption of H* and OH* intermediates, as well as their interaction with the active site for both commercial Pt/C (Fig. 6g) and the Pt/Mo_2_N cluster heterostructured catalyst (Fig. 6h). For commercial Pt/C (wherein the Pt nanoparticles are directly loaded onto the porous carbon substrate), the H_2_O molecules can swiftly adsorb onto the Pt sites depending on the formation of robust Pt–H–OH bonds. However, the lack of active sites for OH activation is not conducive to H-OH dissociation, resulting in sluggish kinetics of the Volmer step and making it a rate-determining step in the alkaline HER process. Besides, the generated Pt–H* could be rapidly consumed through re-association with the abundant OH^−^ in alkaline electrolytes. What’s worse, the Pt-OH* intermediates may block the Pt active sites and hinder the protons transfer and thus adversely affect the HER performance, particularly when operating at a large current density. The electrocatalytic alkaline HER mechanism becomes completely different for our Pt/Mo_2_N-NrGO cluster heterostructured catalyst (wherein the Pt atoms are anchored on the Mo_2_N cluster support). The strongly coupled interface between Pt atoms with moderate H* adsorption energy and the Mo_2_N clusters with OH* affinity may effectively facilitate water dissociation, akin to a pair of scissors. It is worth mentioning that the absorbed Mo-OH* can be migrated to the other Mo sites on surface of the Mo_2_N support in Pt/Mo_2_N and thus to regenerate the interfacial Pt–N-Mo sites. Moreover, the Mo_2_N cluster support offers abundant localized regions of OH* adsorption (Mo–OH), which may restrain the formation of Pt-OH shell and provide a hydrophilic channel for mass transfer. Benefiting from the elaborate co-catalytic Pt/Mo_2_N cluster heterostructure, the activity and stability of alkaline HER have been significantly improved, rendering it a promising candidate for large-scale application.

## Conclusions

In summary, we developed a strongly coupled Pt/Mo_2_N–NrGO cluster heterostructure catalyst for alkaline HER, by using a delicate PtMo6 POMs cluster as a precursor. The strong electrons interaction at the well-defined boundary of the Pt/Mo_2_N cluster heterostructure plays a key role in improving both the activity and durability of the alkaline HER. Both experimental results and DFT calculations demonstrate that the abundant interfacial Pt–N–Mo bonds within the Pt/Mo_2_N–NrGO catalyst significantly improve the H-OH bond cleavage and facilitate effective proton/electron transfers to the Pt active sites, fundamentally altering the HER kinetics to be similar to acidic Tafel step limited kinetics. The cooperative effect of the Pt-Mo_2_N interface, which enhances the water dissociation kinetics and accelerates the Volmer step, has been directly observed via the Operando Raman spectroscopy. This shows that the Mo–OH and Pt–H bonds become progressively stronger during the alkaline HER, indicating the strongly coupled interface between Pt atoms and the Mo_2_N clusters may effectively facilitate water dissociation. More importantly, the as-formed Mo–OH layer may serve as a hydrophilic channel to continually support delivery of reactants (H_2_O and proton) to the Pt active sites, similar to how natural enzymes function by providing a specific local chemical environment or channel for mass transfer. Benefiting from the strongly coupled cluster heterostructure, the optimized Pt/Mo_2_N–NrGO exhibits remarkable HER electrocatalytic activity and stability in alkaline conditions. Notably the Pt/Mo_2_N–NrGO catalyst can sustain an industrial current density of 1.5 A cm^−2^ for more than 500 h under intermittent conditions in a practical PEMWE cell. These benefits led to the overall H_2_ production cost of only $2.02 kg_H2_^−1^ in a 1 MW plant during 30 years, meeting the DOE’s targets of $2 kg_H2_^−1^ ~ $2.5 kg_H2_^−1^. Such strongly coupled cluster heterostructure with a co-catalytic effect design may provide valuable insights for developing highly efficient electrocatalysts for other fundamentally important electrochemical reactions.

## Supplementary Information

Below is the link to the electronic supplementary material.

Supplementary file1 (DOCX 18688 KB)
