# Supplementary material for: A Strongly Coupled Cluster Heterostructure with Pt–N-Mo Bonding for Durable and Efficient H2 Evolution in Anion-Exchange Membrane Water Electrolyzers
Source: Nanomicro Lett. 2025 Jun 13;17:296. doi: 10.1007/s40820-025-01798-x (PMC12165922; doi:10.1007/s40820-025-01798-x)
Supplement: Supplementary file 1 — Supplementary file1 (DOCX 18688 KB) [file 40820_2025_1798_MOESM1_ESM.docx]

Supporting Information for

**A Strongly Coupled Cluster Heterostructure with Pt-N-Mo Bonding for Durable and Efficient H_2_ Evolution in Anion-Exchange Membrane Water Electrolyzers**

Wenbo Zhou ^1, 2, †^, Yichao Huang^1,†,*^, Hanqing Cai^1^, Tao Wang^1^, Haitao Li^1^, Chao Zhang^2^, Lianming Zhao^1, *^, Lulu Chen^1^, Meihong Liao^4^, Zhiqing Tang^1^, Kai Chen^3^, Jing Gu^6^, Wenpei Gao^2, *^, Zhuangjun Fan^1, 5, *^, Zhenhai Wen^3, *^

^1^ State Key Laboratory of Chemical Safety, Shandong Key Laboratory of Intelligent Energy Materials, School of Materials Science and Engineering, China University of Petroleum (East China), Qingdao 266580, P. R. China

^2^ State Key Laboratory of Metal Matrix Composites, School of Materials Science and Engineering, Future Material Innovation Center, Zhangjiang Institute for Advanced Study, Shanghai Jiao Tong University, Shanghai 200240, P. R. China

^3^ Fujian Institute of Research on the Structure of Matter, Chinese Academy of Science, Institute of Materials, Fuzhou 350002, P. R. China

^4^ School of Mechanical and Electronic Engineering, Qingdao Binhai University, Qingdao 266555, P. R. China

^5^ Institute of Energy, Hefei Comprehensive National Science Center, Hefei 230051, P. R. China

^6^ Department of Chemistry and Biochemistry, San Diego State University, 5500 Campanile Drive, San Diego, California 92182-1030, USA

† Wenbo Zhou and Yichao Huang contributed equally to this work.

* Corresponding authors. E-mails: [yichaoh@upc.edu.cn](mailto:yichaoh@upc.edu.cn) (Yichao Huang); [Lmzhao@upc.edu.cn](mailto:Lmzhao@upc.edu.cn) (Lianming Zhao); [gaowenpei@sjtu.edu.cn](mailto:gaowenpei@sjtu.edu.cn) (Wenpei Gao); [fanzhj666@163.com](mailto:fanzhj666@163.com) (Zhuangjun Fan); [wen@fjirsm.ac.cn](mailto:wen@fjirsm.ac.cn) (Zhenhai Wen)

**Supplementary Figures and Tables**


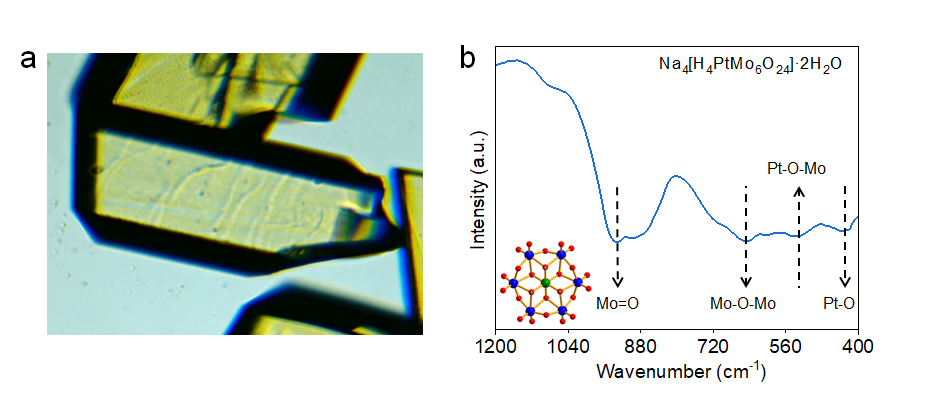


**Fig. S1** **a**) Crystallographic morphology and **b**) Fourier transform infrared spectroscopy (FT-IR) of Na_4_[H_4_PtMo_6_O_24_]·2H_2_O (PtMo6), the peaks of Mo=O (933 cm^-1^), Mo-O-Mo (661 cm^-1^), Pt-O-Mo (518 cm^-1^) and Pt-O (424 cm^-1^) verify the successful synthesis of PtMo6 compound (the inset is the structure diagram of PtMo6) [S1]


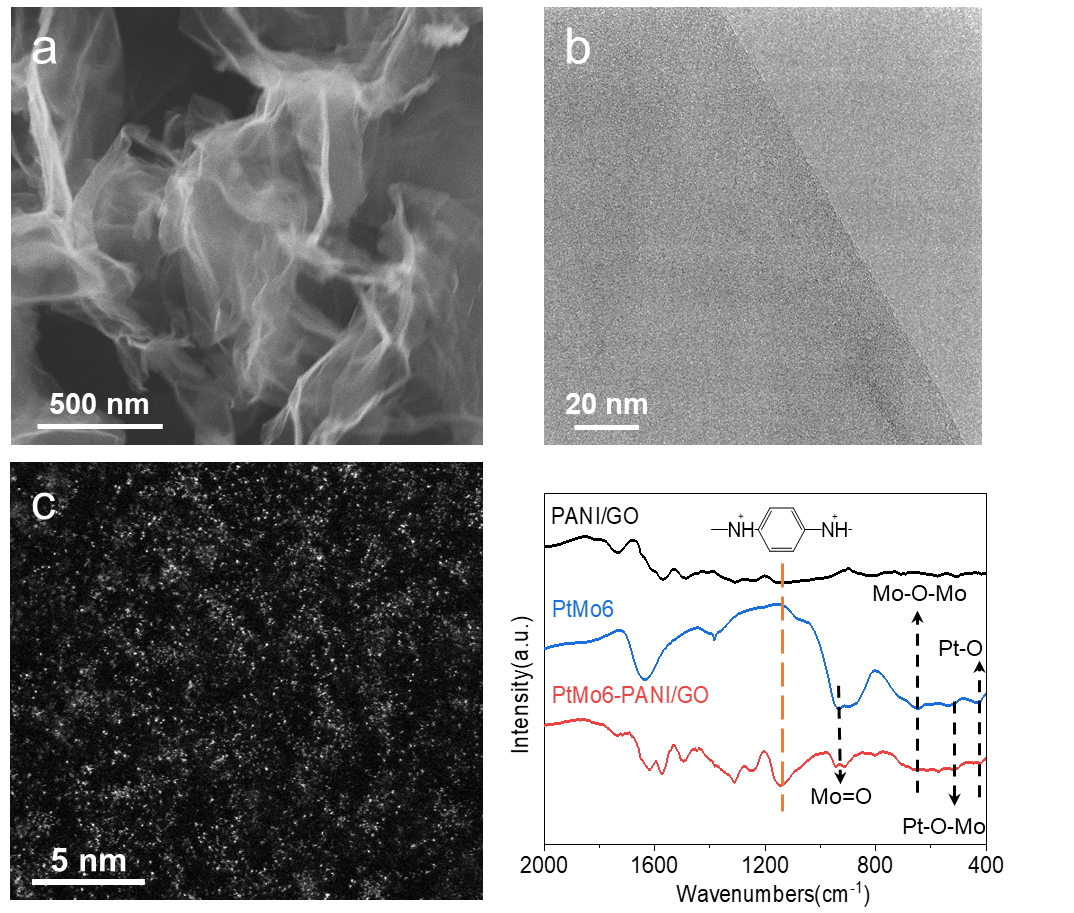


**Fig. S2** **a**) SEM, **b**) TEM, and **c**) HAADF-STEM spectra of PtMo6-PANI/GO precursor. The results confirm the PtMo6 POMs cluster is uniformly dispersed on the PANI/GO matrix without significant agglomeration. **d**) Fourier transform infrared (FT-IR) spectra of PtMo6-PANI/GO, PANI/GO, and PtMo6 [S1]


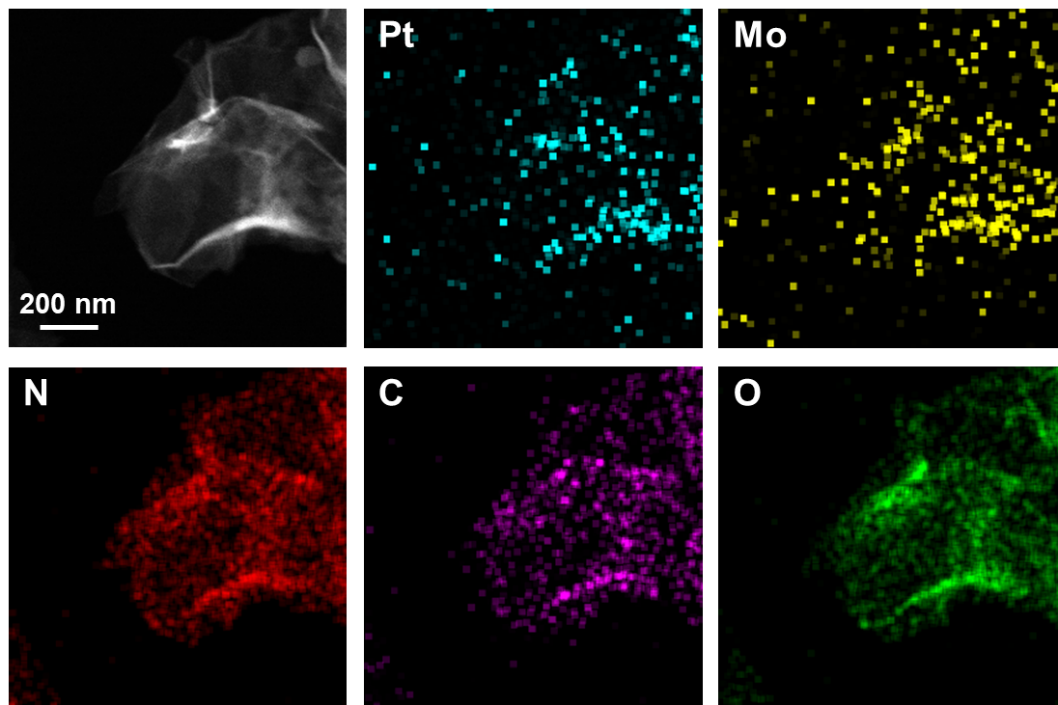


**Fig. S3** The energy dispersive X-ray spectroscopy (EDS) mapping of Pt (cyan), Mo (yellow), N (red), C (purple) and O (green) in the PtMo6-PANI/GO nanosheet. The EDS mapping images show the homogeneous dispersion of Pt and Mo in the PtMo6-PANI/GO nanosheets, further confirming the successful combination of PtMo6 POMs cluster and PANI/GO substrate.


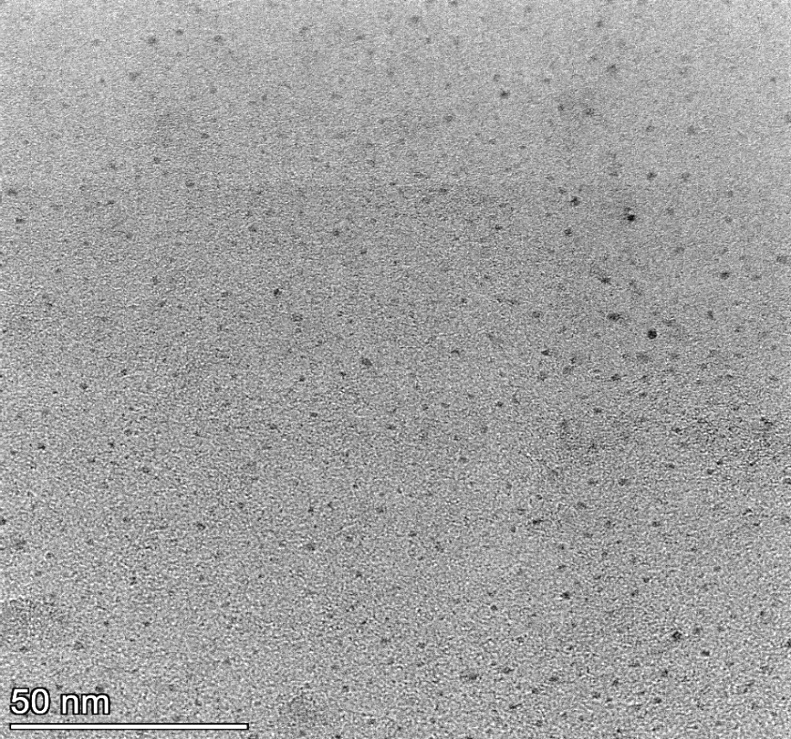


**20 nm**


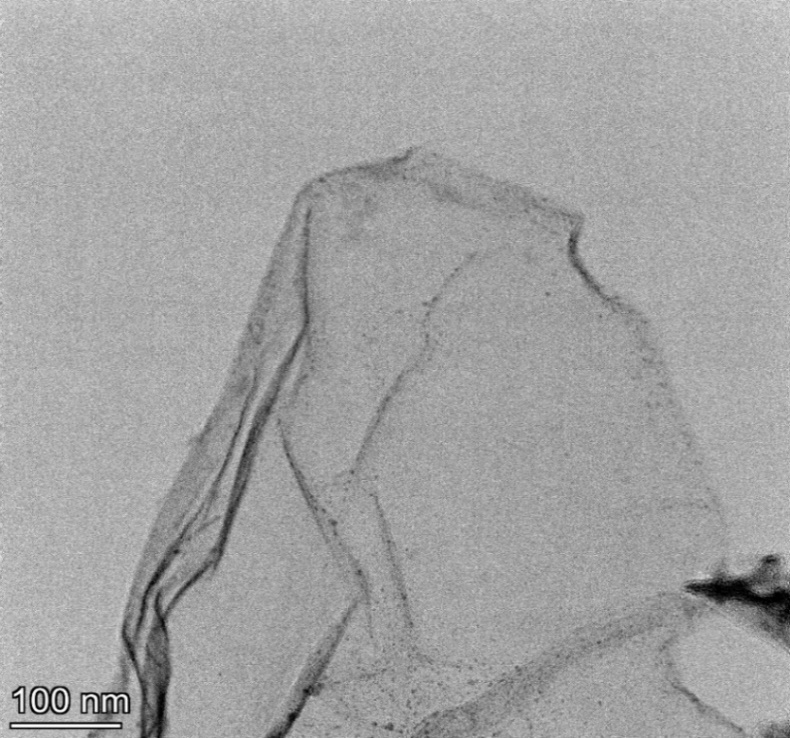


**100 nm**


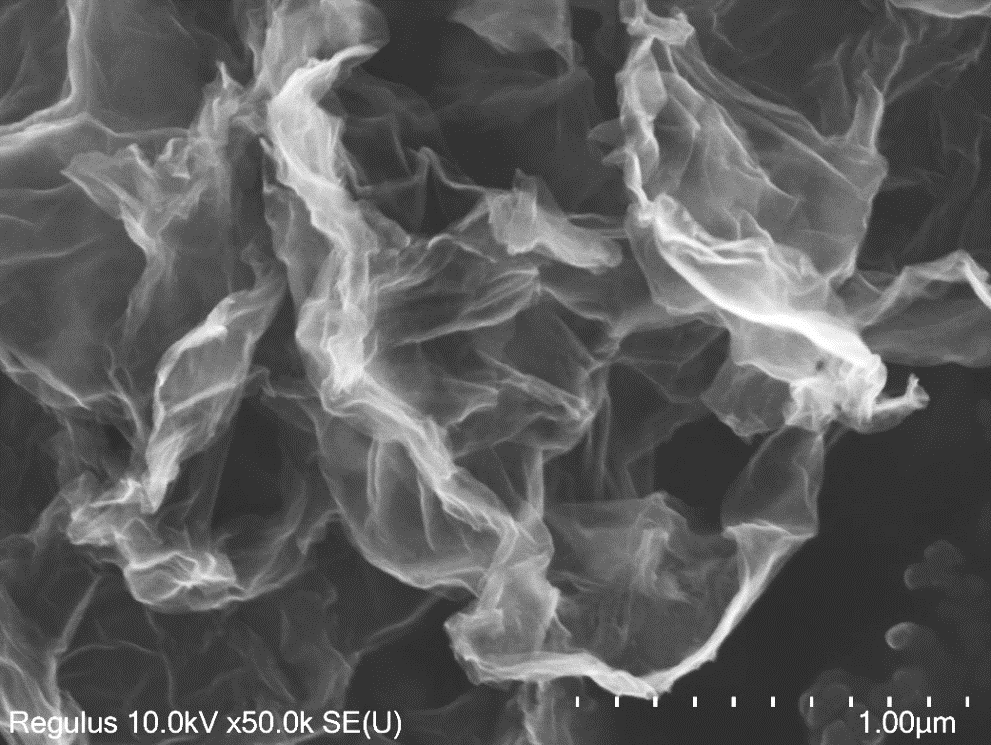


**500 nm**


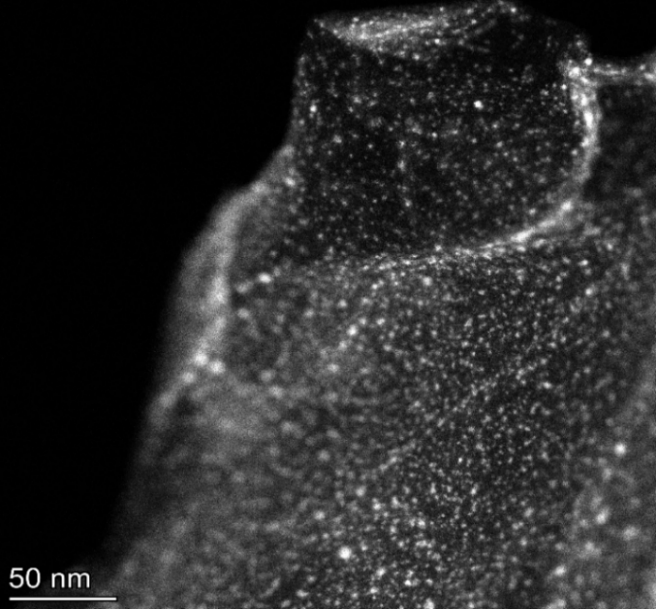


**50 nm**

a

b

c

d

**Fig. S4** **a**) The SEM, **b**) TEM and **c**) HRTEM (the inset is particle size distribution) and **d**) HAADF-STEM images of Pt/Mo_2_N-NrGO, demonstrating that the quantum dots with an average size of 2.0 nm are uniformly loaded on the NrGO surface. It is noteworthy that achieving high-density dispersion of such small-size nanoparticles is remarkably surprising.


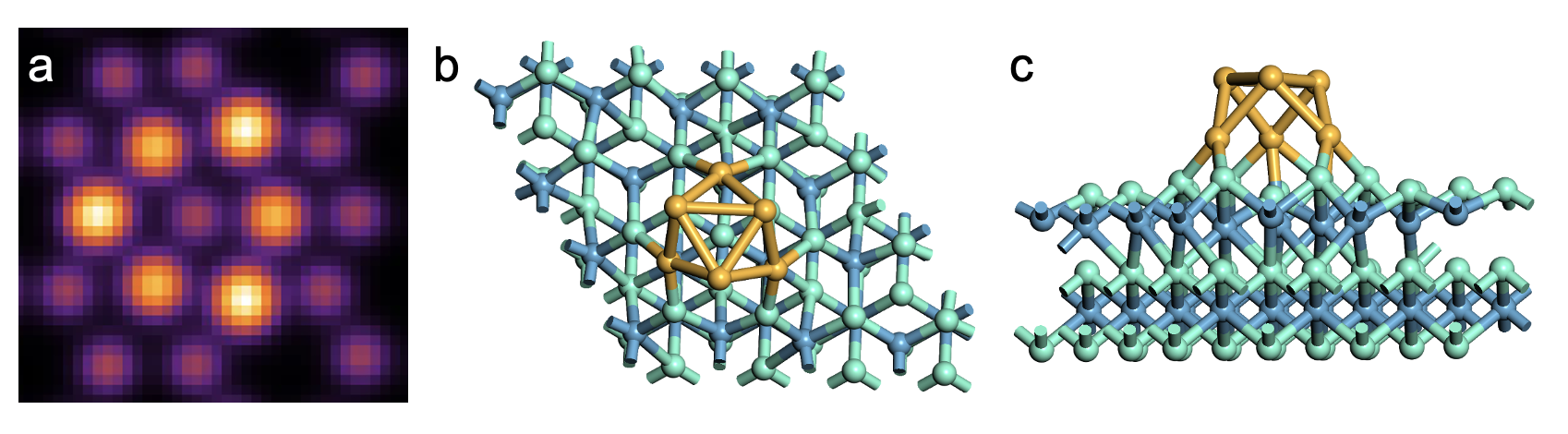


**Fig. S5** **a**) The HAADF simulation used Dr. probe and **b~c**) atomic model of the DFT-derived structure of Pt/Mo_2_N, including top view and side view


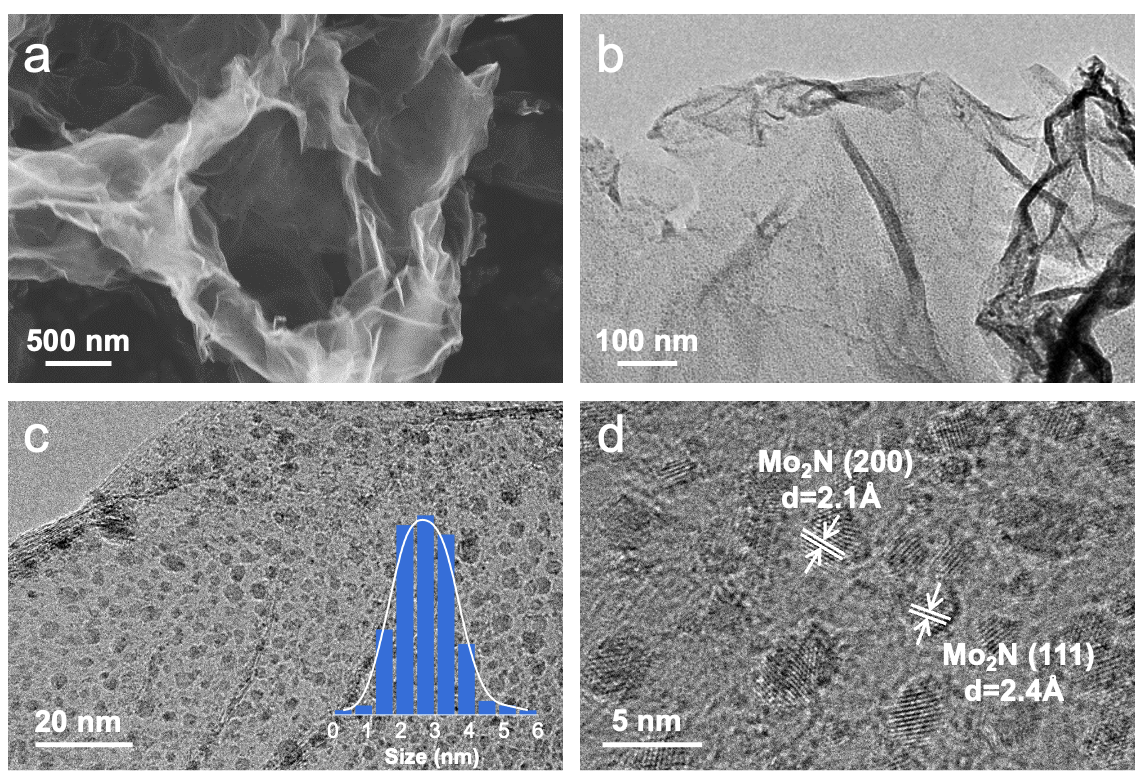


**Fig. S6** **a**) SEM, **b~c**) TEM (the inset image is particle size distribution) and **d**) HRTEM images of Mo_2_N-NrGO. The hexagonal Mo_2_N quantum dots with an average size of 2.72 nm, exposed (111) and (200) crystalline surface, are loaded at NrGO nanosheets


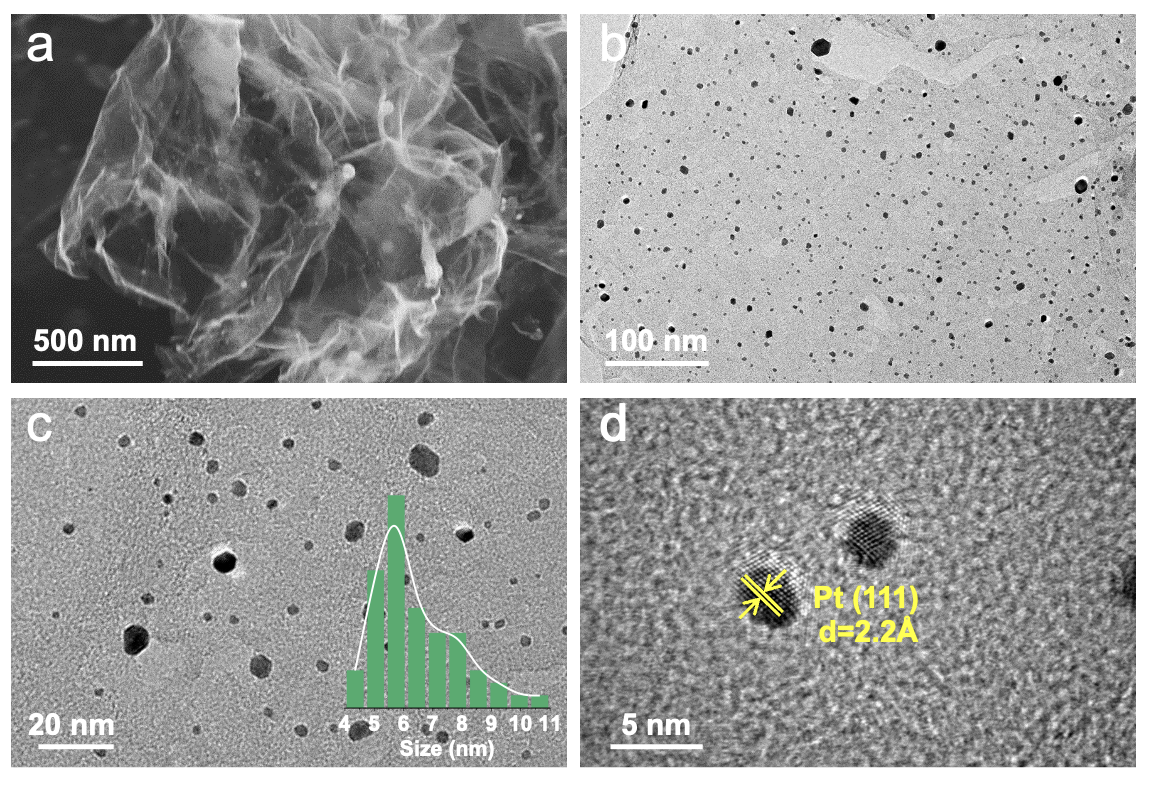


**Fig. S7** **a**) SEM, **b~c**) TEM (the inset image is particle size distribution) and **d**) HRTEM images of Pt-NrGO. Without Mo_2_N support, the Pt atoms undergo an Ostwald ripening process, resulting in severe agglomeration and sintering into Pt nanoparticles with an average size of 5.75 nm that expose the (111) crystalline surface


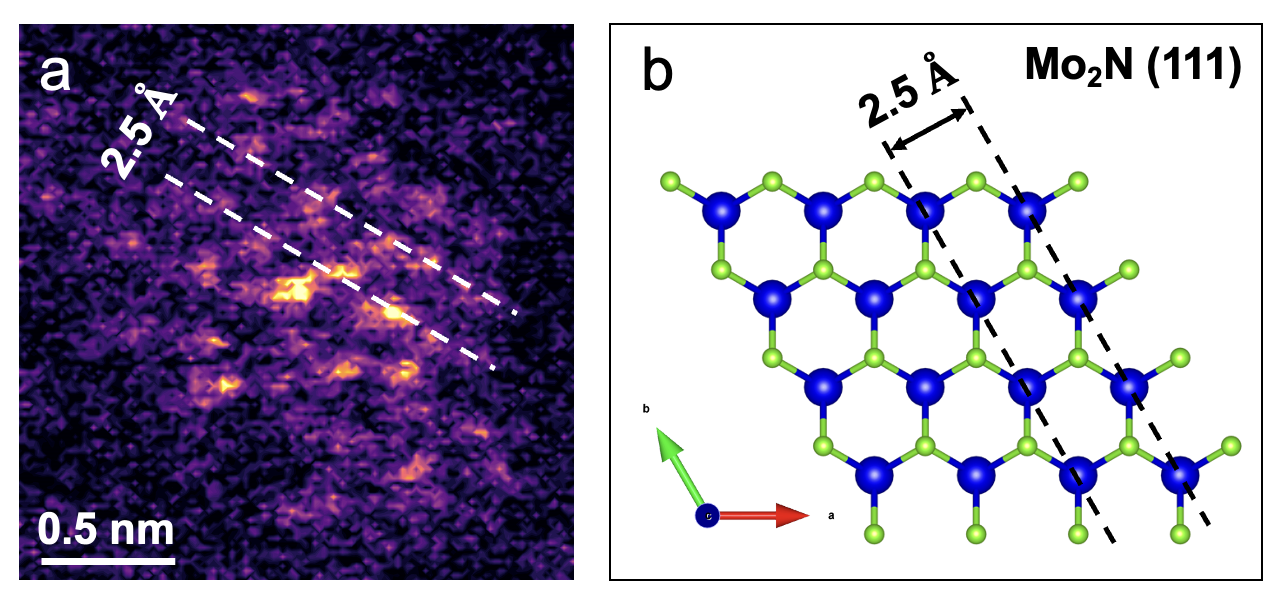


**Fig. S8** **a**) The HAADF-STEM image of Pt/Mo_2_N-NrGO, where the bright spots in the center represent Pt atoms and the surrounding darker spots represent Mo atoms due to the different contrast of Pt and Mo. The crystal plane spacing of Mo_2_N is 0.25 nm, which is consistent with that of the **b**) crystal model of Mo_2_N (111)


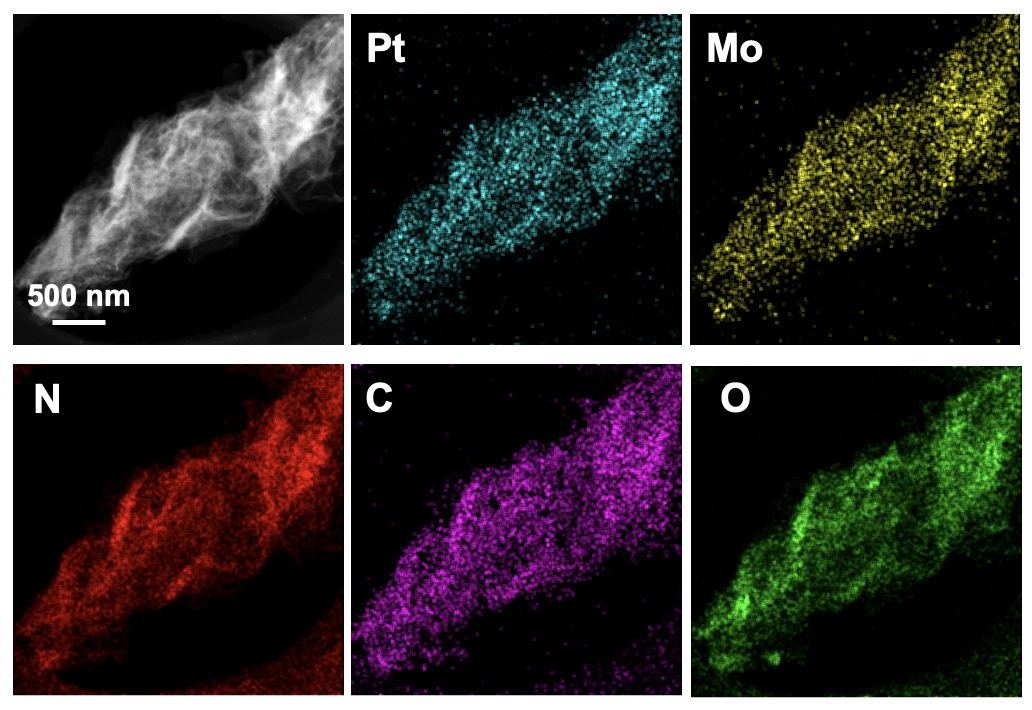


**Fig. S9** STEM-EDS element mapping of Pt (cyan), Mo (yellow), N (red), C (purple) and O (green) in the Pt/Mo_2_N-NrGO under scale bar of 500 nm


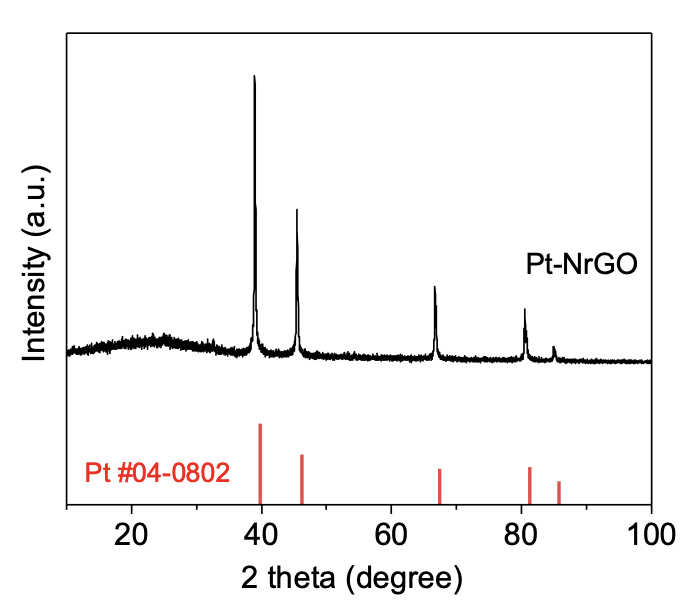


**Fig. S10** XRD pattern of Pt-NrGO with sharp peaks of Pt


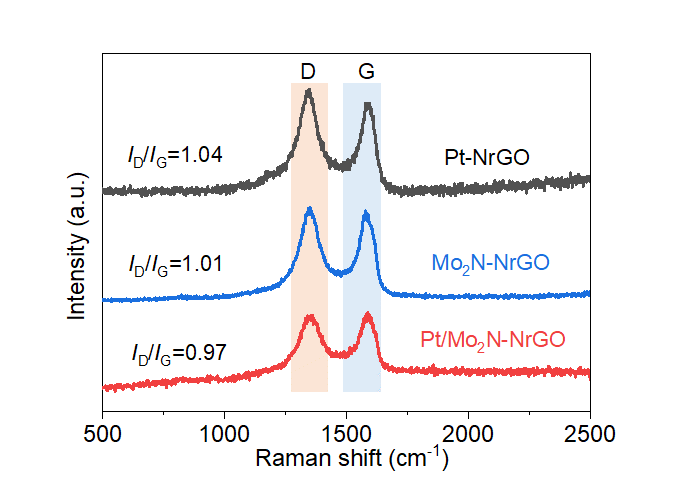


**Fig. S11** Raman spectroscopy of Pt/Mo_2_N-NrGO, Pt-NrGO and Mo_2_N-NrGO where the D band (1350 cm^-1^) and G band (1580 cm^-1^) provide the evidence of defects and crystallinity of graphene structure, respectively


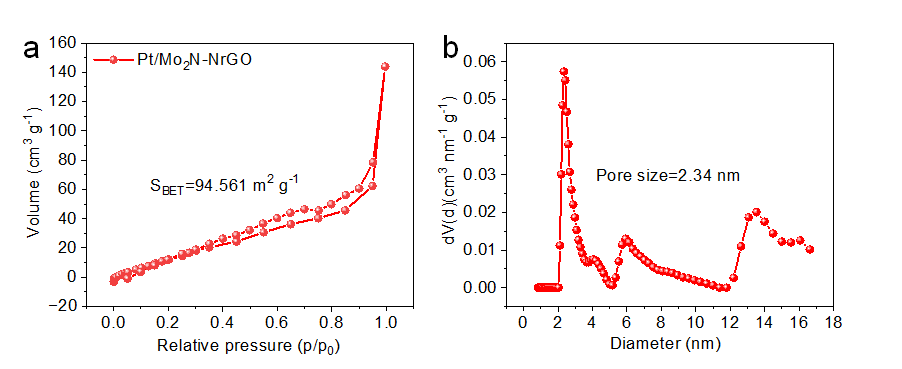


**Fig. S12** **a**) The N_2_ adsorption-desorption isotherm of Pt/Mo_2_N-NrGO measured by the Brunauer-Emmett-Teller (BET) method. **b**) Hole size distribution of Pt/Mo_2_N-NrGO used the Barrett-Joyner-Hallender method. The specific surface area and average pore size of Pt/Mo_2_N rGO are 94.56 m_2_·g^-1^ and 2.34 nm


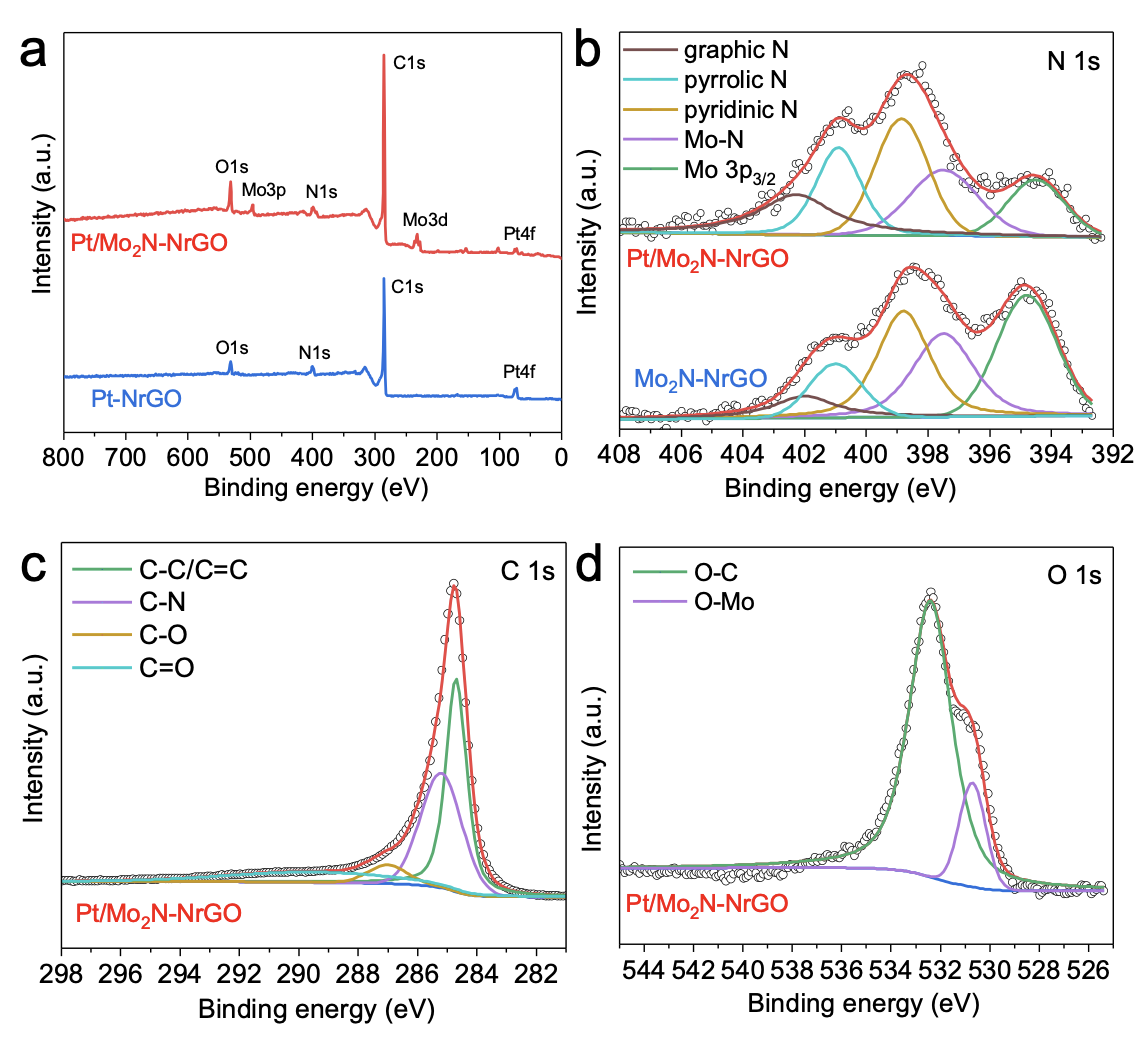


**Fig. S13** **a**) The XPS survey spectrum of Pt/Mo_2_N-NrGO and Pt-NrGO. **b**) High-resolution XPS signals of N 1s for Pt/Mo_2_N-NrGO and Pt-NrGO. **c~d**) High-resolution XPS signals of C 1s and O 1s for Pt/Mo_2_N-NrGO

With regard to N 1s (**Fig.** S11b), the four peaks at 397.48 eV, 398.88 eV, 400.88 eV and 402.38 eV are derived from N-Mo, pyridinic-N, pyrrolic-N and graphic-N, respectively. The main peak of the C 1s spectrum is located at 284.68 eV in **Fig.** S11c, demonstrating the existence of the NrGO matrix. The O 1s spectrum is deconstructed into two peaks at 532.48 eV and 530.68 eV, assigned to O-C and O-Mo, respectively (**Fig.** S11d).


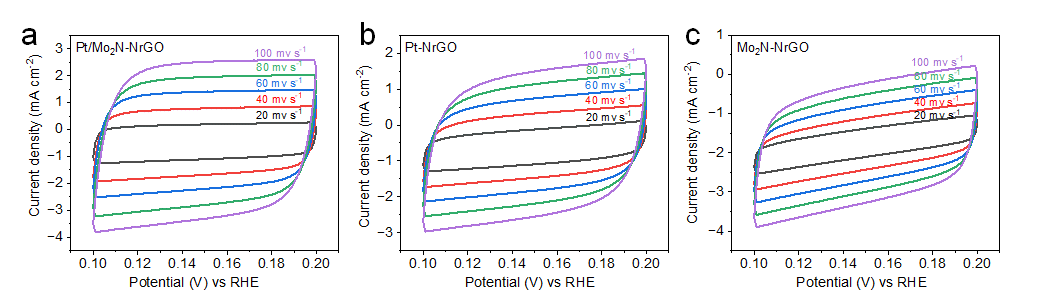


**Fig. S14** CVs of **a**) Pt/Mo_2_N-NrGO, **b**) Pt-NrGO and **c**) Mo_2_N-NrGO with different rates from 20 to 100 mV s^−1^


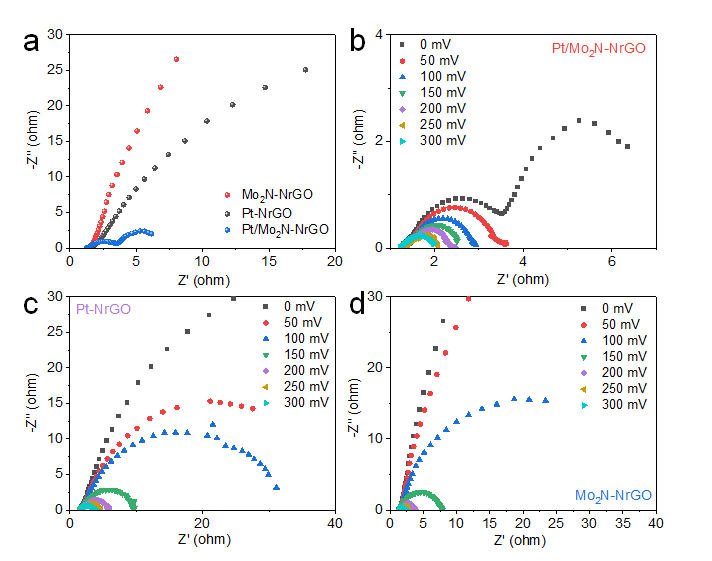


**Fig. S15** **a**) EIS spectra of Pt/Mo_2_N-NrGO, Pt-NrGO and Mo_2_N-NrGO at open circuit voltage. The Nyquist plots of **b**) Pt/Mo_2_N-NrGO, **c**) Pt-NrGO and **d**) Mo_2_N-NrGO at different overpotentials of 0, 50, 100, 150, 200, 250 and 300 Mv


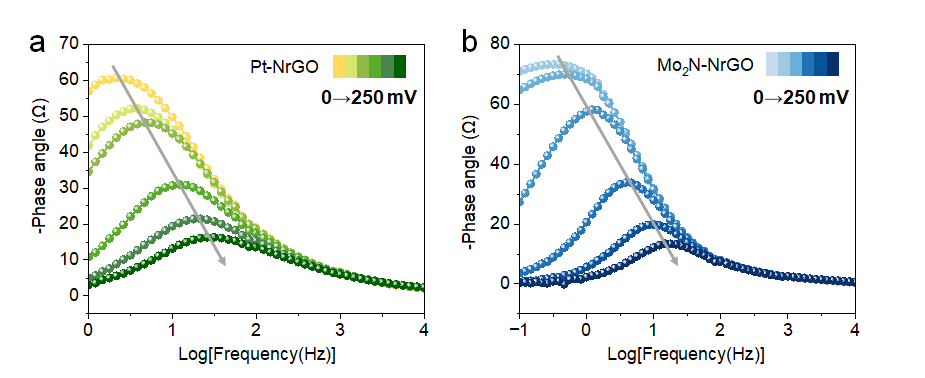


**Fig. S16** Bode phase plot of **a**) Pt-NrGO and **b**) Mo_2_N-NrGO with increasing overpotentials from 0 mV to 250 mV


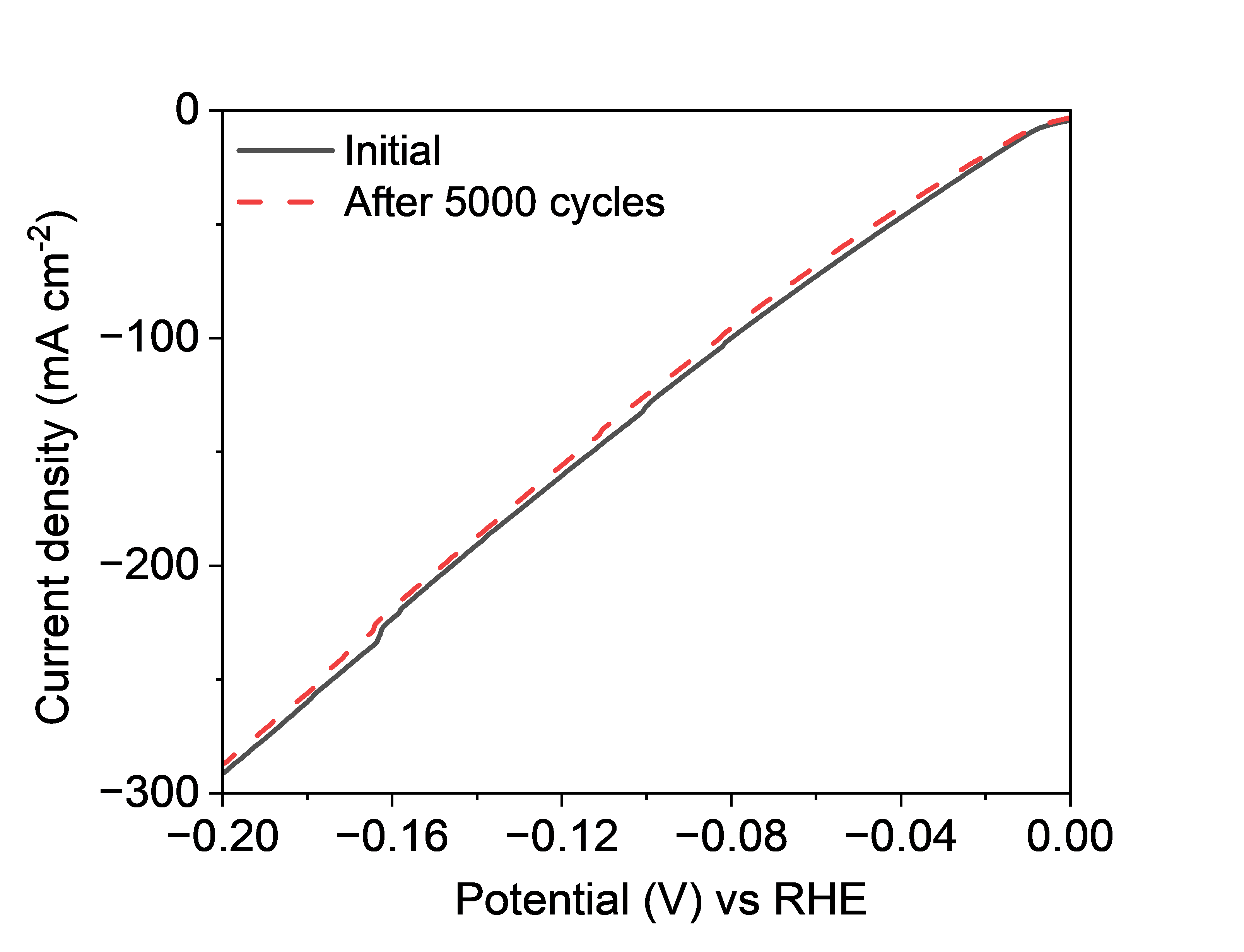


**Fig. S17** The polarization curves of Pt/Mo_2_N-NrGO after 5000 CV cycles from 0.0 V to -0.5 V (RHE) in 1.0 M KOH with a scan rate of 5 mV s^-1^


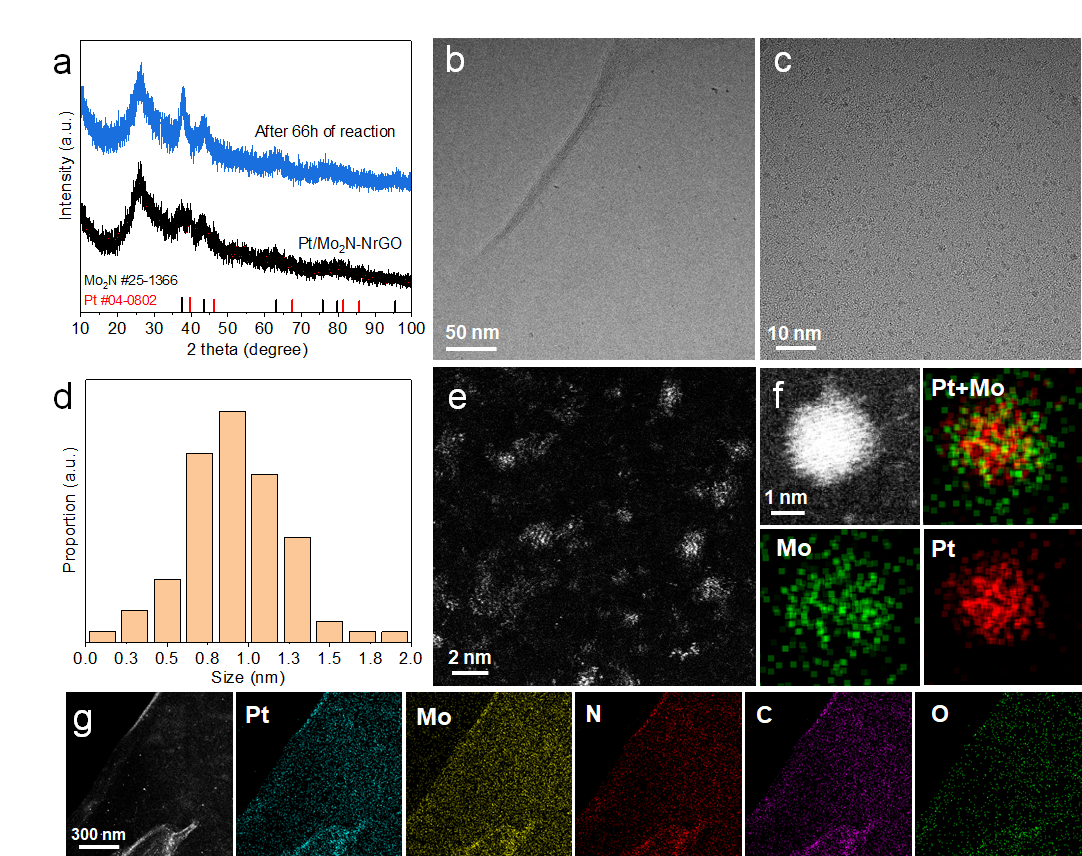


**Fig. S18** The phase, morphology, and atomic distribution of Pt/Mo_2_N-NrGO after stability test at 230 mA·cm^-2^, for 66 h in 1 M KOH. **a**) XRD pattern obverses the characteristic peaks of Mo_2_N without obvious peaks of Pt, indicating that the Pt/Mo_2_N-NrGO have excellent phase structure stability. **b**) TEM and **c**) HRTEM show the Pt/Mo_2_N quantum dots with **d**) an average size of 0.94 nm uniformly anchored on the NrGO matrix without aggregation**. e**) The HAADF-STEM image is consistent with **Fig.** 2a in the article. **f**) The magnified HAADF-STEM image and STEM-EDS mappings of a single nanocluster indicate that Pt atoms are anchored on the Mo_2_N. **g**) The STEM-EDS mappings of Pt (cyan), Mo (yellow), N (red), C (purple), and O (green) show the uniform distribution of Pt/Mo_2_N quantum dots.

The excellent stability of our Pt/Mo_2_N-NrGO co-catalyst can be ascribed to the intimate contact between Pt atoms and Mo_2_N clusters through strong metal-support interaction, as well as the strong combination of Pt/Mo_2_N clusters to NrGO by metal-nitrogen/carbon chemical bonding.


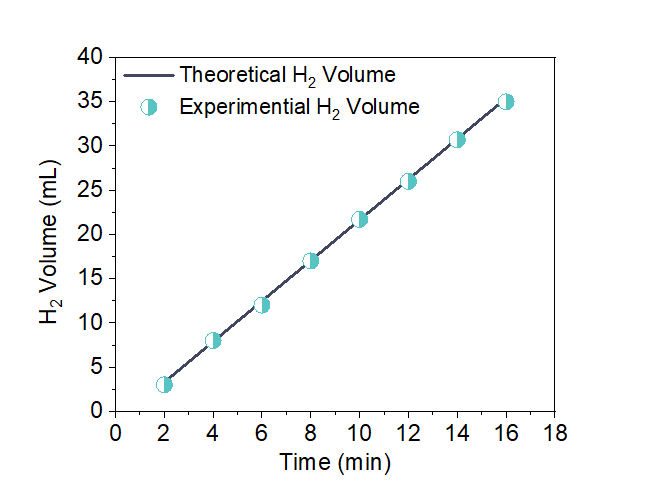


**Fig. S19** The Faradaic efficient testing of Pt/Mo_2_N-NrGO. The amount of H_2_ was theoretically calculated (100%) and experimentally measured (99.87%) versus time on the electrocatalyst in mA·cm^-2^


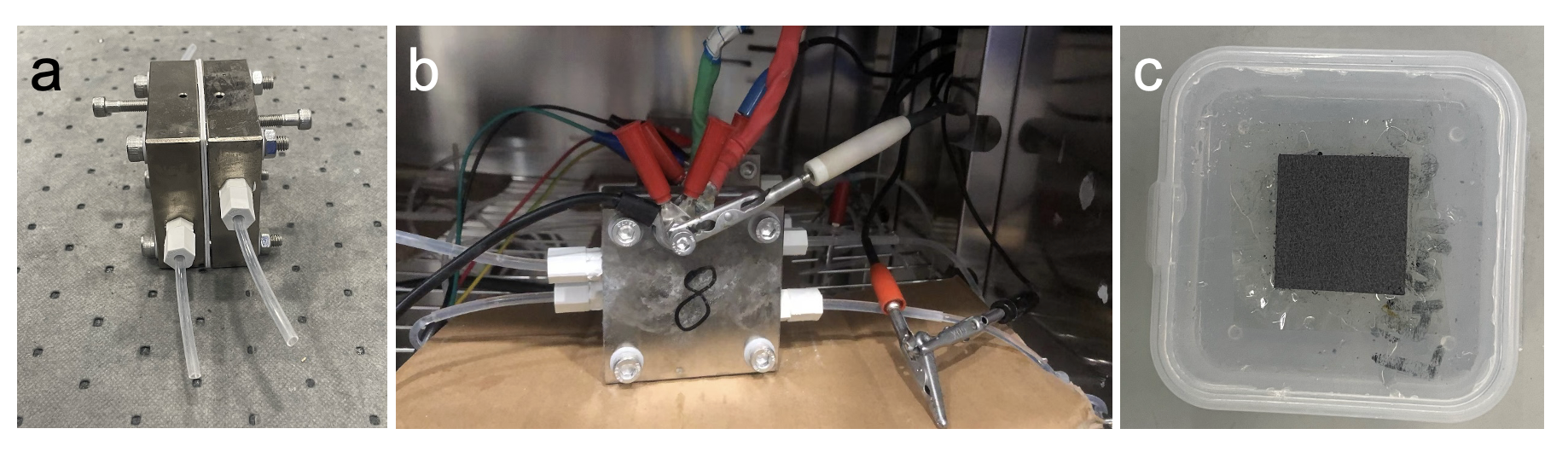


**Fig. S20** **a**) and **b**) Image of our AEMWE electrolyzer. c) The image of the AEM membrane electrode

**Technology-economy analysis:**

A TEA analysis was conducted to estimate the CAPEX, OPEX and the resulting LOCH when scaling up our lab-scale product to 1MW industrialization, considering various operating conductions. The all calculations were conducted under the assumption of complete performance retention from the lab-scale tests to plant-scale, as well as the linear relationship between manufacturing costs per single and plant scale. The CAPEX of a generic 1 MW-scale AEMWE system was estimated based on the unitary cost of the diaphragm/electrode package (DEP), considering cost of raw materials of electrodes and the associate manufacture, providing by IRENA reports. The annual CAPEX was subsequently derived by factoring in the depreciation of the total CAPEX through a capital recovery factor (CRF). Besides, the OPEX-related calculations have been performed by the current density and associate voltage collected from our single AEMWE cell. In addition, we also estimated other operational expenses including process water consumption, labor costs, maintenance expenditures and miscellaneous ancillary charges, except for the electrical energy supplied to the electrolyzer. The production of H_2_ of ideal AEMWE single cell per year was calculated using the Faraday’s law:

Annual H_2_ production = $\frac{I\times t\times FE\times MM(H2)}{n\times F}$ (S1)

where I was the total current output of the plant per year, t was the time, FE was the Faradaic Efficiency, MM_H2_ was the molecular mass of H_2_ (g·mol^-1^), n was the number of electrons transferred during the generation of H_2_ molecule and F was the Faradaic constant.

Finally, the LCOH of annual H_2_ production was calculated as:

LCOH ($·kg_H2_^-1^) = $\frac{Annual CAPEX+Annual OPEX}{Annual H2 production}$ (S2)

where detailed CAPEX, OPEX and Annual H_2_ production analysis was available in the section of technology-economy analysis of supporting information.

Details see the followings:

When scaling our single-cell device to an initial 1 MW-scale plant, we roughly estimate the LCOH of an AEMWE electrolyzer based on the average CAPEX, as published by IRENA (shown in the following Figure) [S2], and basic OPEX such as water and electricity supplied to the electrolyzer and other ancillary costs.


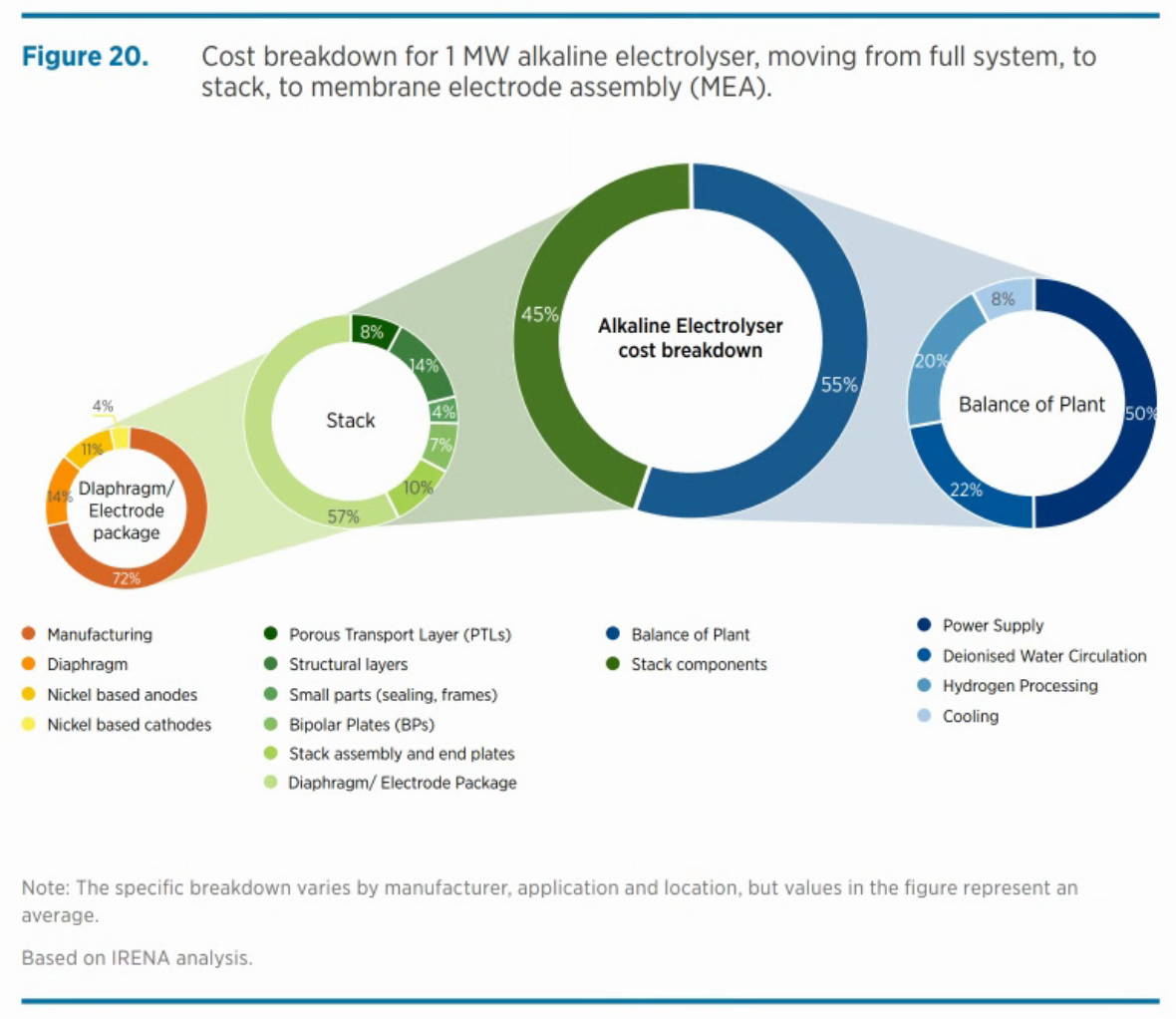


1. The unitary cost ($ per 1 cm^2^) of a diaphragm/electrode package (DEP) includes the cost of the cathode, anode, and diaphragm as follows (the catalyst load is 1.5 mg·cm^-2^ and the area of the cathode and anode is 4 cm^2^):

The cost of raw materials of cathode:

|  | Platinum^1^ | Molybdenum^2^ | Graphene Oxide^3^ | Carbon paper^4^ |
| --- | --- | --- | --- | --- |
| Loading (ug·cm^-2^) | 45.0 | 324.0 | 1113.0 | - |
| Cost ($) | 0.01224 | 0.00024 | 0.00385 | 0.0132 |

1 https://www.leyan.com/12325-31-4.html (cost of Na_2_Pt(OH)_6_ is 0.068 $·mg^-1^)

2 http://www.macklin.cn/products/A915090 (cost of H_24_Mo_7_N_6_O_24_·4H_2_O is 0.000178 $·mg^-1^)

3 https://www.aladdin-e.com/zh_cn/g139805.html

4 https://m.tb.cn/h.5vI20LicJbcSvdN tk=QSTCWmHsnxt

The cost of raw materials of synthetic routes:

|  |  | Volume (uL) | Mass (mg) | Cost ($) |
| --- | --- | --- | --- | --- |
| Synthesis of PtMo6 | HNO_3_^1^ | 30.0 |  | 0.00019 |
| Synthesis of PtMo6-PANI-GO | Aniline^2^ | 3.25 |  | 0.00164 |
|  | H_2_O_2_^3^ | 2.77 |  | 0.00025 |
|  | HCl^4^ | 0.67 |  | 0.00032 |
| Synthesis of Pt@Mo_2_N-NrGO | Urea^5^ |  | 3.3 | 0.00005 |

1 https://www.reagent.com.cn/goodsDetail/Nitric-acid/%E7%A1%9D%E9%85%B8/d17ba41c5d3d469a94d684b93cfd372e

2 https://www.energy-chemical.com/front/search_goodsbyclass.htm keyword=%E8%8B%AF%E8%83%BA

3 https://www.reagent.com.cn/goodsDetail/Hydrogen-peroxide-30%25-aqueous-solution/%E8%BF%87%E6%B0%A7%E5%8C%96%E6%B0%A230%25/e9b7901600624cba9e2d3f9413b0b40b

4 https://www.reagent.com.cn/goodsDetail/Hydrochloric-acid/%E7%9B%90%E9%85%B8/f3048cdd35044454869c9b959f0b98de

5 https://www.aladdin-e.com/zh_cn/u111897.html

Cost of cathode, anode and diaphragm per 1 cm^2^:

|  | Cathode^1^ | Anode^2^ | Diaphragm^3^ |
| --- | --- | --- | --- |
| Cost ($) | 0.00799 | 0.02 | 0.038 |

1 Cost of cathode = cost of raw materials of cathode + cost of raw materials of synthetic routes

2 NiFe LDH is from Adv. Funct. Mater. 2024, 34, 2310762

3 https://doi.org/10.1021/jacs.3c06726

2. Standard CAPEX of single AEMWE cell: The average cost per 1 MW stack components is estimated as 270 $·kW^-1^ from previous literature [S3]. Therefore, the CAPEX of an ideal 1 MW AEMWE system is 600000 $ calculated as following:

CAPEX of ideal 1 MW AEMWE = $\frac{270}{45}\times$100$\times$1000

Therefore, the cost breakdown from IRENA is showing following:

|  | | Proportion (%) | Actual cost ($·MW^-1^) |  | Proportion in stack components/BOP (%) | Actual cost ($·MW^-1^) |  | Proportion in DEP (%) | Actual cost ($·MW^-1^) |
| --- | --- | --- | --- | --- | --- | --- | --- | --- | --- |
| Stack components | 45 | 270000 | PTLs | 8 | 21600 |  |  |  |  |
|  |  |  | Structural layers | 14 | 37800 |  |  |  |  |
|  |  |  | Small parts | 4 | 10800 |  |  |  |  |
|  |  |  | BPs | 7 | 18900 |  |  |  |  |
|  |  |  | Stack assembly and end plates | 10 | 27000 |  |  |  |  |
|  |  |  | DEP | 57 | 153900 | Cell manufacturing | 72 | 110808 |  |
|  |  |  |  |  |  | Diaphragm | 14 | 21546 |  |
|  |  |  |  |  |  | Anode | 11 | 16929 |  |
|  |  |  |  |  |  | Cathode | 4 | 6156 |  |
| BOP | 55 | 330000 | Power supply | 50 | 165000 |  |  |  |  |
|  |  |  | DI circulation | 22 | 72600 |  |  |  |  |
|  |  |  | H2 processing | 20 | 66000 |  |  |  |  |
|  |  |  | Cooling | 8 | 26400 |  |  |  |  |

The basic parameter of 1 MW AEMWE cell:

| Cell voltage | 1.8 V |
| --- | --- |
| Current density | 1 A·cm^-2^ |
| Electrode area | 700 cm^-2^ |
| Cell power | 1.8 W·cm^-2^ |
|  | 1260 W |
| Cells per stack | 200 units |
| Stacks in the system | 5 units |
| Gross system power | 1.26 MW |
| Stack (cell) efficiency | 82 % at Higher heating value^1^ |
| Stack power | 1.03 MW |

1 Higher heating value (HHV) is from IRENA.

3. The cost breakdown of our cell stacks:

| Electrode area | 700 cm^-2^ |
| --- | --- |
| Cost of cathodel | 5.6 $·cm^-1^ |
| Cost of anode | 14 $·cm^-1^ |
| Cost of diaphragm | 26.6 $·cm^-1^ |

Our DEP is calculated as following:

|  | | Proportion in stack components/BOP (%) | | Actual cost ($·MW^-1^) | |  | | Proportion in DEP (%) | | Actual cost ($·MW^-1^) | |
| --- | --- | --- | --- | --- | --- | --- | --- | --- | --- | --- | --- |
| DEP | | 58.2 | | 157006 | | Cell manufacturing | | 70.576 | | 110.808 | |
|  |  |  |  |  |  | Diaphragm | | 16.942 | | 26.600 | |
|  |  |  |  |  |  | Anode | | 8.917 | | 14.000 | |
|  |  |  |  |  |  | Cathode | | 3.565 | | 5.598 | |

4. The capital recovery factor (CRF) has been estimated according to the equation:

CRF = $\frac{i \times\left( 1+i \right)^{n}}{\left( 1+i \right)^{n}-1}$ = 0.061

where i is the discount rate (set it as 4.5%) and n is the AEMWE plant lifetime (set is as 30 years).

5. CAPEX and OPEX calculated: The CAPEX is calculated based electrochemical data on the Higher Heating Value (HHV = 141.7 kJ·g_H2_^-1^) and Lower Heating Value (LHV = 120.0 kJ·g_H2_^-1^).

The electrochemical data are shown as following:

| operative conditions | Current density (mA cm^-2^) | Cell voltage (V) | Single cell power (W) | Desired system net power (MW) | Energy Efficiency (%, HHV) | Required system gross power (MW) | Cells required to meet the ideal 1 MW-scale, HHV (net system power) | Overall electricity fed to the AEL, HHV (A) | Energy Efficiency (%, LHV) | Required system gross power LHV (MW) | Cells required to meet the ideal 1 MW-scale, LHV (net system power) | Overall electricity fed to the AEL, LHV (A) |
| --- | --- | --- | --- | --- | --- | --- | --- | --- | --- | --- | --- | --- |
| 1 | 118.025 | 1.4599 | 120.6132883 | 1 | 99.4022222 | 0.986171682 | 8176.310388 | 675506.3234 | 85.87344154 | 1.164504394 | 9654.859849 | 797660.3836 |
| 2 | 180.525 | 1.4799 | 187.0112633 | 1 | 99.0318293 | 0.999681808 | 5345.570051 | 675506.3234 | 84.71291121 | 1.180457602 | 6312.227302 | 797660.3836 |
| 3 | 245.475 | 1.4999 | 257.7315668 | 1 | 98.69798267 | 1.013191935 | 3931.190685 | 675506.3234 | 83.58333042 | 1.196410809 | 4642.081001 | 797660.3836 |
| 4 | 319.425 | 1.52 | 339.8682 | 1 | 97.39283172 | 1.026769612 | 3021.081736 | 675506.3234 | 82.47805086 | 1.212443783 | 3567.394016 | 797660.3836 |
| 5 | 394.925 | 1.54 | 425.72915 | 1 | 96.12798975 | 1.040279738 | 2443.524805 | 675506.3234 | 81.40690734 | 1.228396991 | 2885.39554 | 797660.3836 |
| 6 | 472.675 | 1.5599 | 516.1280128 | 1 | 94.90166306 | 1.053722314 | 2041.591016 | 675506.3234 | 80.36838086 | 1.244270432 | 2410.778725 | 797660.3836 |
| 7 | 560.725 | 1.5799 | 620.1225993 | 1 | 93.70030015 | 1.06723244 | 1721.002334 | 675506.3234 | 79.35099519 | 1.26022364 | 2032.216922 | 797660.3836 |
| 8 | 641.525 | 1.5999 | 718.4630933 | 1 | 92.52897319 | 1.080742567 | 1504.242288 | 675506.3234 | 78.35904575 | 1.276176848 | 1776.259434 | 797660.3836 |
| 9 | 738.575 | 1.6199 | 837.4923498 | 1 | 91.38656967 | 1.094252693 | 1306.582315 | 675506.3234 | 77.39159041 | 1.292130055 | 1542.855951 | 797660.3836 |
| 10 | 828.3 | 1.6399 | 950.830419 | 1 | 90.27203135 | 1.10776282 | 1165.047728 | 675506.3234 | 76.44773297 | 1.308083263 | 1375.727193 | 797660.3836 |
| 11 | 1027.825 | 1.6799 | 1208.650252 | 1 | 88.12256933 | 1.134783073 | 938.8845703 | 675506.3234 | 74.6274405 | 1.339989678 | 1108.666197 | 797660.3836 |
| 12 | 1132.875 | 1.6999 | 1348.041949 | 1 | 87.08577223 | 1.148293199 | 851.8230462 | 675506.3234 | 73.74941897 | 1.355942886 | 1005.861047 | 797660.3836 |
| 13 | 1343.3 | 1.7399 | 1636.045369 | 1 | 85.08368539 | 1.175313452 | 718.3868335 | 675506.3234 | 72.05393258 | 1.387849301 | 848.2951193 | 797660.3836 |
| 14 | 1458.175 | 1.7599 | 1796.369528 | 1 | 84.11677039 | 1.188823579 | 661.7923318 | 675506.3234 | 71.23509137 | 1.403802509 | 781.4664452 | 797660.3836 |
| 15 | 1687.2 | 1.7999 | 2125.753896 | 1 | 82.24740497 | 1.215843832 | 571.9588866 | 675506.3234 | 69.65200139 | 1.435708924 | 675.3881186 | 797660.3836 |
| 16 | 1794.925 | 1.8199 | 2286.608805 | 1 | 81.34353767 | 1.229353958 | 537.6319531 | 675506.3234 | 68.88655272 | 1.451662132 | 634.8537313 | 797660.3836 |
| 17 | 2032.475 | 1.8599 | 2646.140177 | 1 | 79.59412023 | 1.256374211 | 474.7950324 | 675506.3234 | 67.40504183 | 1.483568547 | 560.6538008 | 797660.3836 |
| 18 | 2179.1 | 1.8799 | 2867.543063 | 1 | 78.74732923 | 1.269884337 | 442.8475212 | 675506.3234 | 66.68792877 | 1.499521755 | 522.9291146 | 797660.3836 |
| 19 | 2403.3 | 1.9199 | 3229.866969 | 1 | 77.10667442 | 1.29690459 | 401.5349867 | 675506.3234 | 65.29852456 | 1.53142817 | 474.1458968 | 797660.3836 |
| 20 | 2592.225 | 1.94 | 3520.24155 | 1 | 76.30778568 | 1.310482267 | 372.2705527 | 675506.3234 | 64.62197799 | 1.547461144 | 439.5894777 | 797660.3836 |
| 21 | 2742.15 | 1.96 | 3762.2298 | 1 | 75.5291348 | 1.323992394 | 351.9169387 | 675506.3234 | 63.96257005 | 1.563414352 | 415.5552518 | 797660.3836 |
| 22 | 2832.6 | 1.9799 | 3925.785318 | 1 | 74.76999051 | 1.33743497 | 340.6795995 | 675506.3234 | 63.31968145 | 1.579287793 | 402.2858271 | 797660.3836 |
| 23 | 3031.175 | 1.9999 | 4243.432818 | 1 | 74.02225322 | 1.350945096 | 318.3613726 | 675506.3234 | 62.68645297 | 1.595241001 | 375.9317208 | 797660.3836 |

Total CAPEX is calculated as the function:

Total CAPEX = $\frac{i \times\left( 1+i \right)^{n}}{\left( 1+i \right)^{n}-1}$ = 0.061=Single cell CAPEX of DEP $\times$ Cells required to meet the ideal 1MW-scale + ideal actual cost of stack components except for DEP + BOP

The total and annual CAPEX on HHV and LHV are calculated as following:

| Operative conditions | Total CAPEX of HHV ($) | Annual CAPEX of HHV ($ year^-1^) | Total CAPEX of LHV ($) | Annual CAPEX of LHV ($ year^-1^) |
| --- | --- | --- | --- | --- |
| 1 | 1729827.269 | 106196.765 | 1961967.95 | 120448.2396 |
| 2 | 1285384.924 | 78911.76372 | 1437155.614 | 88229.20057 |
| 3 | 1063319.313 | 65278.81324 | 1174933.139 | 72130.95822 |
| 4 | 920427.028 | 56506.43538 | 1006201.166 | 61772.24203 |
| 5 | 829747.3024 | 50939.46712 | 899123.523 | 55198.58034 |
| 6 | 766641.4099 | 47065.29901 | 824605.9815 | 50623.8335 |
| 7 | 716307.162 | 43975.20187 | 765169.6238 | 46974.94379 |
| 8 | 682274.601 | 41885.89044 | 724982.8414 | 44507.81521 |
| 9 | 651240.8603 | 39980.68122 | 688337.1659 | 42258.08066 |
| 10 | 629019.1246 | 38616.45458 | 662096.9996 | 40647.15636 |
| 11 | 593510.2215 | 36436.50823 | 620166.9032 | 38073.00305 |
| 12 | 579841.0667 | 35597.33773 | 604025.9096 | 37082.08255 |
| 13 | 558890.8218 | 34311.16987 | 579287.1621 | 35563.33267 |
| 14 | 550005.1629 | 33765.66556 | 568794.6799 | 34919.18299 |
| 15 | 535900.8007 | 32899.777 | 552139.7788 | 33896.71292 |
| 16 | 530511.2767 | 32568.90581 | 545775.6493 | 33506.00919 |
| 17 | 520645.5225 | 31963.23194 | 534125.8379 | 32790.80929 |
| 18 | 515629.5814 | 31655.29557 | 528202.8474 | 32427.18777 |
| 19 | 509143.2784 | 31257.09142 | 520543.6045 | 31956.97503 |
| 20 | 504548.5957 | 30975.01676 | 515118.0501 | 31623.89187 |
| 21 | 501352.9624 | 30778.83191 | 511344.5398 | 31392.23026 |
| 22 | 499588.6362 | 30670.5172 | 509261.1646 | 31264.32864 |
| 23 | 496084.5475 | 30455.39579 | 505123.4199 | 31010.30611 |

The OPEX includes the main operating expenses i.e., the electricity fed to the AEMWE, the process water consumed, labor, maintenance and ancillary costs, is calculated according to the following equations:

OPEX_electricity_:

I_total_ = i $\times$ A_single cell_ $\times$ n_cells per stack_ $\times$ n_stacks per system_

P_gross_ = I_total_ $\times$ E_cell_ $\times$ t_annual AEMWE operation_

OPEX_electricity_ = P_gross_ $\times$ Celectricity

where I and i indicate the current and current density, A stands for area, E for voltage, P for power, t for time and C for cost, respectively.

OPEX_H2O_:

m_H2O consumed per year_ = m _produced H2 per year_ $\times$ m_H2O consumption per kg_

OPEX_H2O_ = m_H2O consumed per year_ $\times$ C_H2O_

where m indicates mass.

The other costs of labor (0.3% of total CAPEX), maintenance (2.5% of total CAPEX) and other ancillary (1% of total CAPEX) are calculated as percentages of the total CAPEX of the whole system. Therefore, the OPEX is calculated as following:

| HHV | | | | | | LHV | | | | | |
| --- | --- | --- | --- | --- | --- | --- | --- | --- | --- | --- | --- |
| Annual electricity cost ($ year-1) | Labour ($ year-1) | Water-related expenses ($ year-1) | Maintainance ($ year-1) | Other ancillary costs ($ year-1) | Annual OPEX ($ year-1) | Annual electricity cost ($ year-1) | Labour ($ year-1) | Wate-related expenses ($ year-1) | Maintainance ($ year-1) | Other ancillary costs ($ year-1) | Annual OPEX ($ year-1) |
| 165676.8425 | 5189.481807 | 2987.720536 | 43245.68172 | 17298.27269 | 400074.8418 | 195636.7382 | 5885.90385 | 3528 | 49049.19875 | 19619.6795 | 469356.2585 |
| 167946.5438 | 3856.154772 | 2987.720536 | 32134.6231 | 12853.84924 | 387725.4352 | 198316.8771 | 4311.466843 | 3528 | 35928.89036 | 14371.55614 | 454773.6675 |
| 170216.245 | 3189.95794 | 2987.720536 | 26582.98283 | 10633.19313 | 383826.3444 | 200997.016 | 3524.799417 | 3528 | 29373.32848 | 11749.33139 | 450169.4912 |
| 172497.2948 | 2761.281084 | 2987.720536 | 23010.6757 | 9204.27028 | 382958.5371 | 203690.5556 | 3018.603497 | 3528 | 25155.02914 | 10062.01166 | 449144.7554 |
| 174766.996 | 2489.241907 | 2987.720536 | 20743.68256 | 8297.473024 | 384052.11 | 206370.6944 | 2697.370569 | 3528 | 22478.08807 | 8991.23523 | 450436.0828 |
| 177025.3487 | 2299.92423 | 2987.720536 | 19166.03525 | 7666.414099 | 386170.7916 | 209037.4326 | 2473.817945 | 3528 | 20615.14954 | 8246.059815 | 452937.8926 |
| 179295.05 | 2148.921486 | 2987.720536 | 17907.67905 | 7163.07162 | 388797.4927 | 211717.5715 | 2295.508871 | 3528 | 19129.2406 | 7651.696238 | 456039.5888 |
| 181564.7512 | 2046.823803 | 2987.720536 | 17056.86503 | 6822.74601 | 392043.6578 | 214397.7104 | 2174.948524 | 3528 | 18124.57103 | 7249.828414 | 459872.7688 |
| 183834.4525 | 1953.722581 | 2987.720536 | 16281.02151 | 6512.408603 | 395403.7782 | 217077.8493 | 2065.011498 | 3528 | 17208.42915 | 6883.371659 | 463840.5109 |
| 186104.1537 | 1887.057374 | 2987.720536 | 15725.47812 | 6290.191246 | 399098.7547 | 219757.9882 | 1986.290999 | 3528 | 16552.42499 | 6620.969996 | 468203.6624 |
| 190643.5562 | 1780.530665 | 2987.720536 | 14837.75554 | 5935.102215 | 406828.2214 | 225118.266 | 1860.50071 | 3528 | 15504.17258 | 6201.669032 | 477330.8743 |
| 192913.2575 | 1739.5232 | 2987.720536 | 14496.02667 | 5798.410667 | 410848.196 | 227798.4049 | 1812.077729 | 3528 | 15100.64774 | 6040.259096 | 482077.7943 |
| 197452.66 | 1676.672465 | 2987.720536 | 13972.27054 | 5588.908218 | 419130.8917 | 233158.6826 | 1737.861486 | 3528 | 14482.17905 | 5792.871621 | 491858.2774 |
| 199722.3612 | 1650.015489 | 2987.720536 | 13750.12907 | 5500.051629 | 423332.6391 | 235838.8215 | 1706.38404 | 3528 | 14219.867 | 5687.946799 | 496819.8409 |
| 204261.7637 | 1607.702402 | 2987.720536 | 13397.52002 | 5359.008007 | 431875.4784 | 241199.0993 | 1656.419336 | 3528 | 13803.49447 | 5521.397788 | 506907.5102 |
| 206531.4649 | 1591.53383 | 2987.720536 | 13262.78192 | 5305.112767 | 436210.079 | 243879.2382 | 1637.326948 | 3528 | 13644.39123 | 5457.756493 | 512025.9511 |
| 211070.8674 | 1561.936568 | 2987.720536 | 13016.13806 | 5206.455225 | 444913.9853 | 249239.516 | 1602.377514 | 3528 | 13353.14595 | 5341.258379 | 522303.8138 |
| 213340.5687 | 1546.888744 | 2987.720536 | 12890.73954 | 5156.295814 | 449262.782 | 251919.6549 | 1584.608542 | 3528 | 13205.07119 | 5282.028474 | 527439.0179 |
| 217879.9712 | 1527.429835 | 2987.720536 | 12728.58196 | 5091.432784 | 458095.1075 | 257279.9326 | 1561.630814 | 3528 | 13013.59011 | 5205.436045 | 537868.5223 |
| 220161.0209 | 1513.645787 | 2987.720536 | 12613.71489 | 5045.485957 | 462482.609 | 259973.4722 | 1545.35415 | 3528 | 12877.95125 | 5151.180501 | 543049.4303 |
| 222430.7222 | 1504.058887 | 2987.720536 | 12533.82406 | 5013.529624 | 466900.5775 | 262653.6111 | 1534.033619 | 3528 | 12783.61349 | 5113.445398 | 548266.3147 |
| 224689.0749 | 1498.765909 | 2987.720536 | 12489.71591 | 4995.886362 | 471350.2386 | 265320.3493 | 1527.783494 | 3528 | 12731.52911 | 5092.611646 | 553520.6229 |
| 226958.7762 | 1488.253643 | 2987.720536 | 12402.11369 | 4960.845475 | 475756.4857 | 268000.4882 | 1515.37026 | 3528 | 12628.0855 | 5051.234199 | 558723.6663 |

6. Annual H_2_ productivity-1 MW net power is estimated by following function:

Annual H_2_ productivity = $\frac{Total current \times t}{n \times F}$ $\times$ $\frac{FEH2\times MH2}{100}$ ÷ 1000

where t stands for time (1 year=30240000s), n for mole of electrons of H_2_, F for Faraday constant, FE for Faraday efficiency, M_H2_ for molar mass of H_2_, as following.

| Operative conditions | Total current of HHV (A) | Total current of LHV (A) | Annual H_2_ production of HHV (kg· year^-1^) | Annual H_2_ production of LHV (kg· year^-1^) |
| --- | --- | --- | --- | --- |
| 1 | 675506.3234 | 797660.3836 | 213408.6097 | 252000 |
| 2 | 675506.3234 | 797660.3836 | 213408.6097 | 252000 |
| 3 | 675506.3234 | 797660.3836 | 213408.6097 | 252000 |
| 4 | 675506.3234 | 797660.3836 | 213408.6097 | 252000 |
| 5 | 675506.3234 | 797660.3836 | 213408.6097 | 252000 |
| 6 | 675506.3234 | 797660.3836 | 213408.6097 | 252000 |
| 7 | 675506.3234 | 797660.3836 | 213408.6097 | 252000 |
| 8 | 675506.3234 | 797660.3836 | 213408.6097 | 252000 |
| 9 | 675506.3234 | 797660.3836 | 213408.6097 | 252000 |
| 10 | 675506.3234 | 797660.3836 | 213408.6097 | 252000 |
| 11 | 675506.3234 | 797660.3836 | 213408.6097 | 252000 |
| 12 | 675506.3234 | 797660.3836 | 213408.6097 | 252000 |
| 13 | 675506.3234 | 797660.3836 | 213408.6097 | 252000 |
| 14 | 675506.3234 | 797660.3836 | 213408.6097 | 252000 |
| 15 | 675506.3234 | 797660.3836 | 213408.6097 | 252000 |
| 16 | 675506.3234 | 797660.3836 | 213408.6097 | 252000 |
| 17 | 675506.3234 | 797660.3836 | 213408.6097 | 252000 |
| 18 | 675506.3234 | 797660.3836 | 213408.6097 | 252000 |
| 19 | 675506.3234 | 797660.3836 | 213408.6097 | 252000 |
| 20 | 675506.3234 | 797660.3836 | 213408.6097 | 252000 |
| 21 | 675506.3234 | 797660.3836 | 213408.6097 | 252000 |
| 22 | 675506.3234 | 797660.3836 | 213408.6097 | 252000 |
| 23 | 675506.3234 | 797660.3836 | 213408.6097 | 252000 |

7. The levelized cost of H_2_ production (LCOH) is calculated as following equation:

LCOH ($·kg_H2_^-1^) = $\frac{Annual CAPEX+Annual OPEX}{Annual H2 production}$

and the results are represented as following:

| Operative conditions | Current density (mA cm-2) | LCOH of HHV ($·kg_H2_^-1^) | LCOH of LHV ($·kg_H2_^-1^) |
| --- | --- | --- | --- |
| 1 | 118.025 | 2.372311067 | 2.34049404 |
| 2 | 180.525 | 2.186590314 | 2.154773286 |
| 3 | 245.475 | 2.104437859 | 2.072620831 |
| 4 | 319.425 | 2.05926543 | 2.027448403 |
| 5 | 394.925 | 2.038303786 | 2.006486758 |
| 6 | 472.675 | 2.030077845 | 1.998260818 |
| 7 | 560.725 | 2.027906442 | 1.996089415 |
| 8 | 641.525 | 2.033327281 | 2.001510254 |
| 9 | 738.575 | 2.040144772 | 2.008327744 |
| 10 | 828.3 | 2.051066308 | 2.019249281 |
| 11 | 1027.825 | 2.077070509 | 2.045253481 |
| 12 | 1132.875 | 2.091975269 | 2.060158241 |
| 13 | 1343.3 | 2.124759925 | 2.092942897 |
| 14 | 1458.175 | 2.141892519 | 2.110075492 |
| 15 | 1687.2 | 2.177865532 | 2.146048504 |
| 16 | 1794.925 | 2.196626394 | 2.164809366 |
| 17 | 2032.475 | 2.234573468 | 2.202756441 |
| 18 | 2179.1 | 2.25350832 | 2.221691292 |
| 19 | 2403.3 | 2.293029318 | 2.261212291 |
| 20 | 2592.225 | 2.312266719 | 2.280449691 |
| 21 | 2742.15 | 2.332049349 | 2.300232321 |
| 22 | 2832.6 | 2.352392232 | 2.320575204 |
| 23 | 3031.175 | 2.372031204 | 2.340214176 |


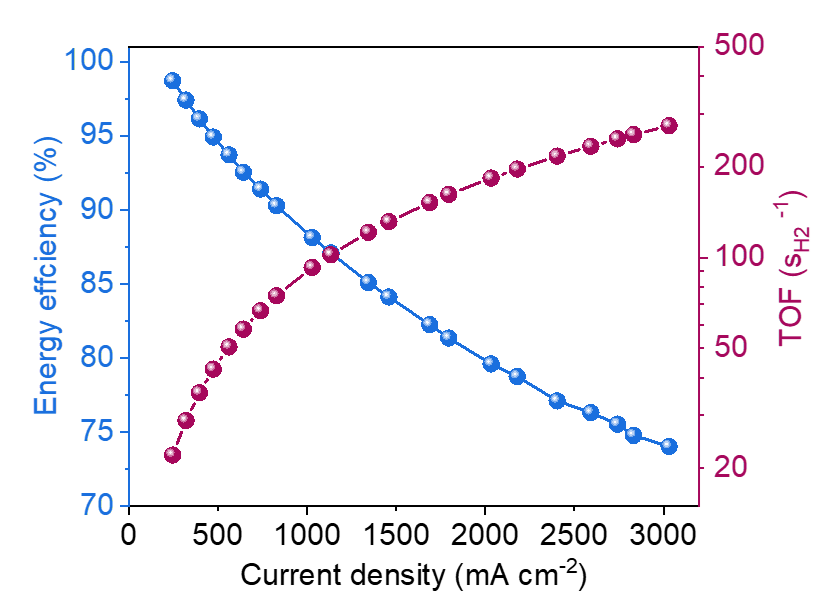


**Fig. S21** H_2_ energy efficiency and TOF values of Pt/Mo_2_N-NrGO||NiFe LDH


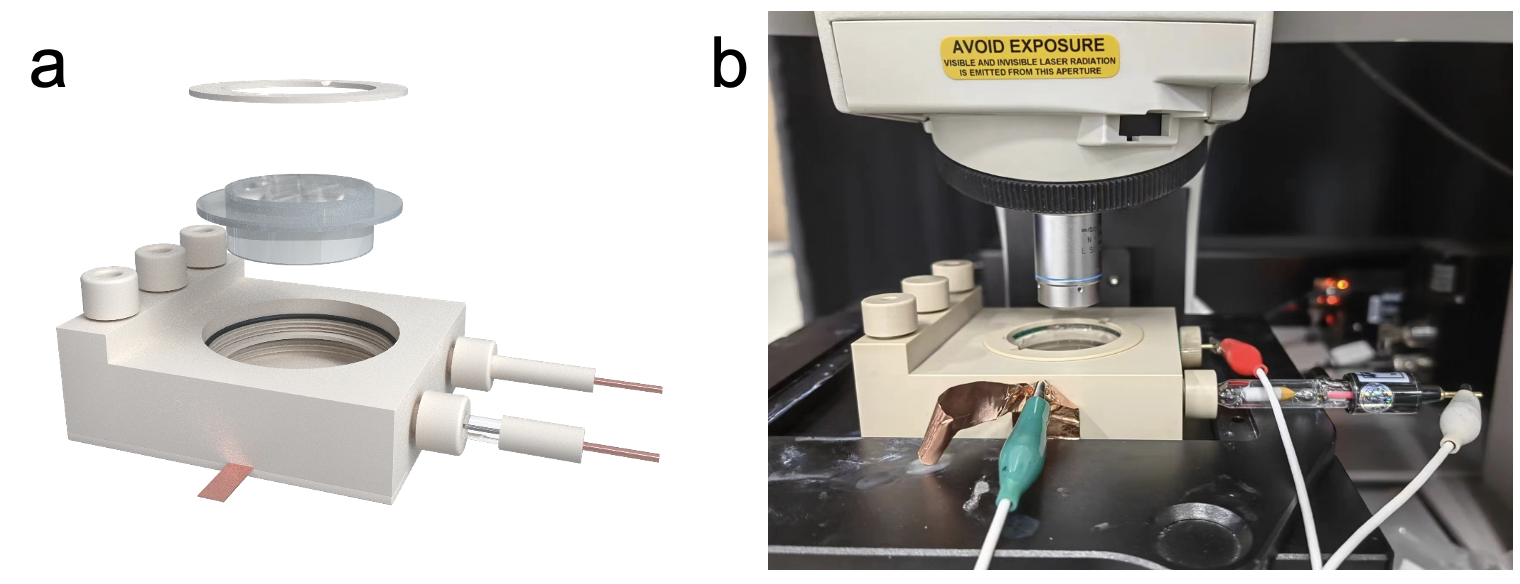


**Fig. S22** **a**) The scheme and **b**) image of in-situ Raman spectroscopy measurement. Pt wire and Hg/HgO are used as counter electrode and (CE) reference electrode, respectively


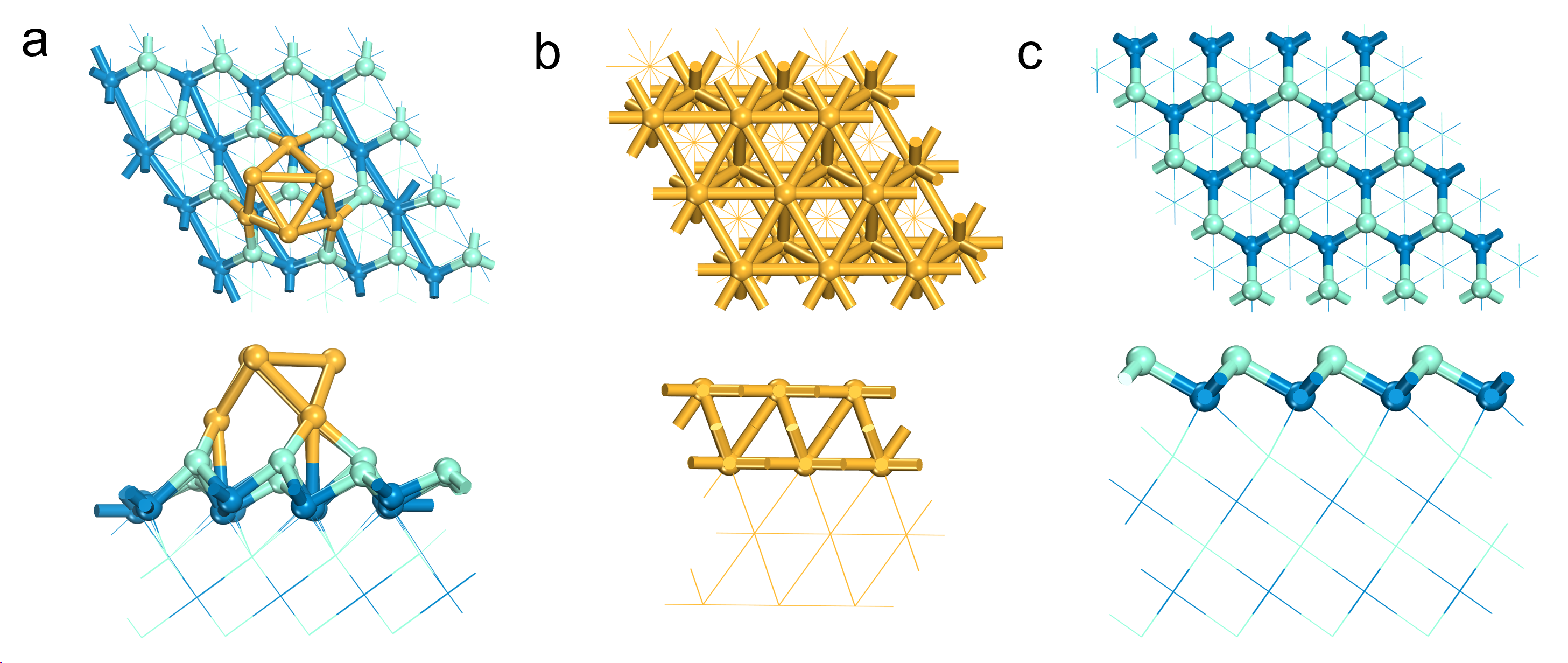


**Fig. S23** The optimized models of **a**) Pt/Mo_2_N, **b**) Pt and **c**) Mo_2_N by DFT relaxation. The blue is Mo atom, the yellow is Pt atom and the green is N atom


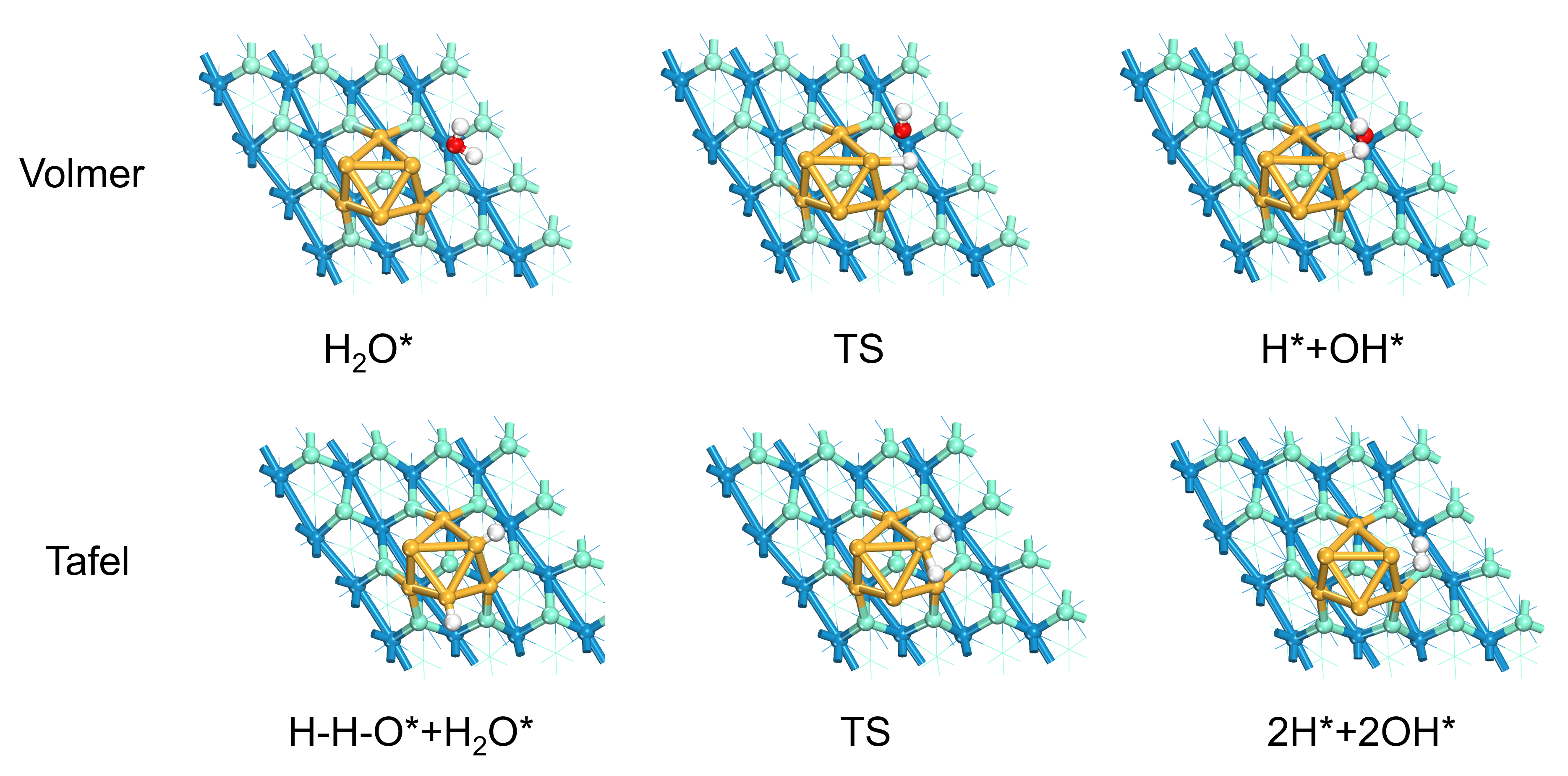


**Fig. S24** Elementary processes of the Volmer and the Tafel mechanism for the HER on Pt/Mo_2_N. The blue, yellow, green, red, and white balls represent the Mo, Pt, N, O, and H atoms, respectively


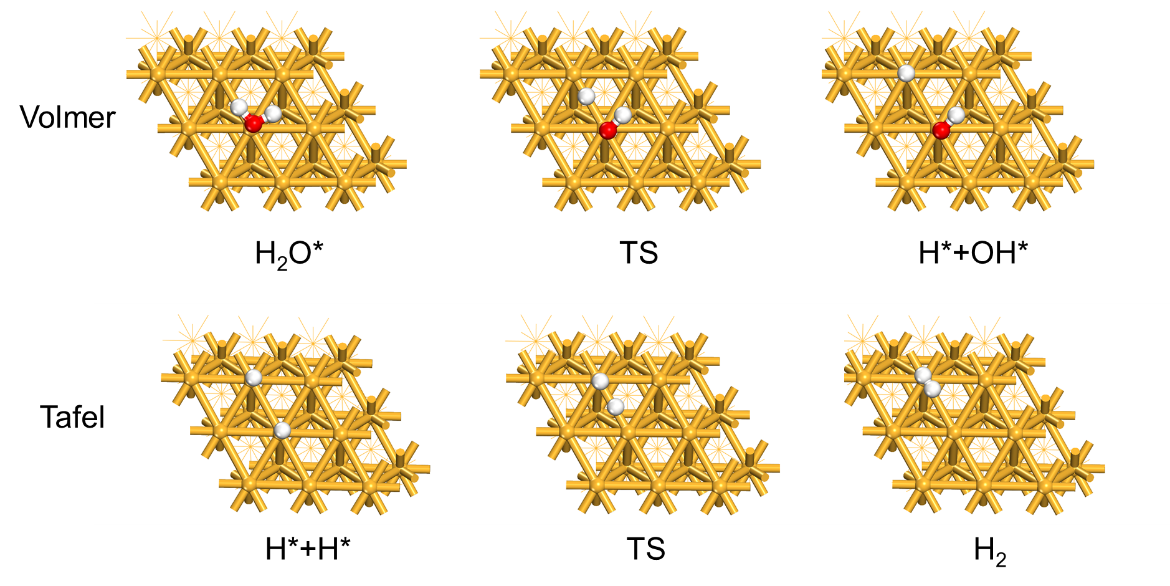


**Fig. S25** Elementary processes of the Volmer and the Tafel mechanism for the HER on Pt. The yellow, red, and white balls represent the Pt, O, and H atoms, respectively


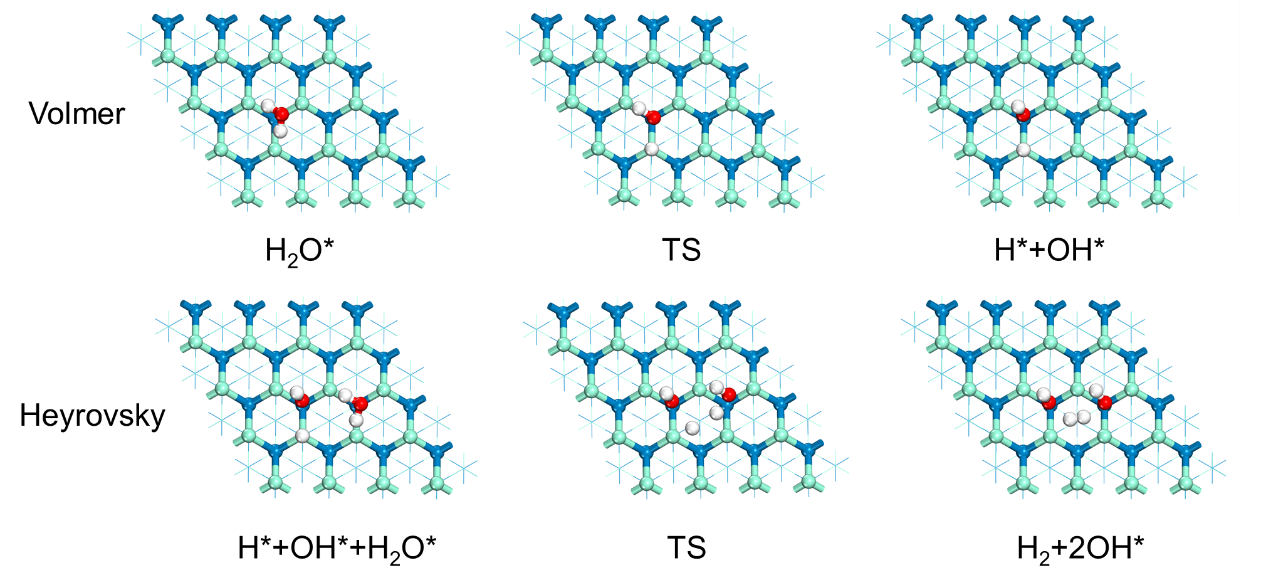


**Fig. S26** Elementary processes of the Volmer and the Tafel mechanism for the HER on Mo_2_N. The blue, green, red, and white balls represent the Mo, N, O, and H atoms, respectively


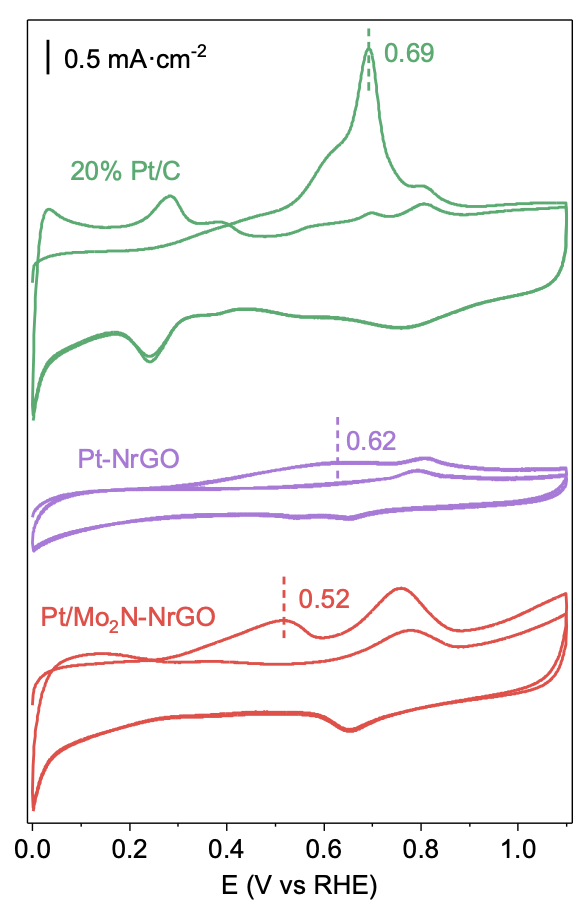


**Fig. S27** The CO stripping voltammetry measurements of commercial Pt/C, Pt-NrGO and Pt/Mo_2_N-NrGO. The dashed lines represent the CO oxidation potentials of above three samples

**Table S1** Summary of elemental analysis of Pt/Mo_2_N-NrGO co-catalysis

| Catalysts | Pt content (wt%) | Mo content (wt%) |
| --- | --- | --- |
| Pt/Mo_2_N-NrGO | 3.394 | 14.923 |

Notes: Data are determined by ICP-AES elemental analysis.

**Table S2** Summary of FWHM, lattice parameters and crystallite sizes of Pt/Mo_2_N-NrGO

|  | Peak positions/ 2 theta | FWHM/2 theta | Lattice parameters | Crystallite sizes/nm |
| --- | --- | --- | --- | --- |
| Mo_2_N of Pt/Mo_2_N-NrGO | 37.30 | 3.76 | (111) | 2.1 |
|  | 42.76 | 3.26 | (200) | 2.5 |
|  | 62.55 | 3.15 | (220) | 2.9 |
| Pt of Pt/Mo_2_N-NrGO | 39.75 | 2.90 | (111) | 2.8 |
|  | 46.16 | 2.56 | (200) | 3.3 |
|  | 67.31 | 2.50 | (220) | 3.8 |
| Mo_2_N-NrGO | 37.57 | 2.41 | (111) | 3.4 |
|  | 42.83 | 2.22 | (200) | 3.8 |
|  | 61.92 | 2.09 | (220) | 4.4 |

**Table S3** Area of XPS results

| Catalysts | Pt^4+^:Pt^0^ | MoOx:Mo-N |
| --- | --- | --- |
| Pt/Mo_2_N-NrGO | 1.16 | 0.59 |
| Pt-NrGO | 0.50 | - |
| Mo_2_N-NrGO | - | 0.65 |

Notes: Data are from **Fig.** 3b, c.

**Table S4** Summary of the HER activity of Pt/Mo_2_N-NrGO, Pt-NrGO, Mo_2_N-NrGO and 20% Pt/C on CP in 1.0 M KOH

| Catalysts | η _onset_ (mV) | η _10_  (mV) | η _100_  (mV) | Tafel slope (mV dec^-1^) | R_s_ ^a)^  (Ω) | R_p_ ^a)^  (Ω) | R_ct_ ^a)^  (Ω) | W_d_ ^a)^ (Ω cm^-2^) | C_dl_ ^b)^  (mF cm^-2^) |
| --- | --- | --- | --- | --- | --- | --- | --- | --- | --- |
| Pt/Mo_2_N-NrGO | ~0 | 11 | 102.2 | 31 | 1.25 | 4.32 | 2.53 | 1.25 | 28.39 |
| Pt/Mo_2_N-NrGO after 5000 CV cycles | ~0 | 12 | 129.5 | 33 |  |  |  |  |  |
| 20%Pt/C | ~0 | 18 | 133 | 39 | - | - | - | - | - |
| Pt-NrGO | ~0 | 32 | 170 | 44 | 14.35 | 107.7 | 86.4 | 1.79 | 19.23 |
| Mo_2_N-NrGO | 15 | 180 | - | 178 | 1.53 | 350.9 | 230.9 | 2.17 | 14.58 |

Notes: EIS Data are measured at η = 0 mV vs. RHE. Notes: Rs, Rp, Rct and W_d_ represent the electrolyte, electrode porosity, charge transfer resistance and Warburg impedance, respectively.

**Table S5** Comparison of HER performance for Pt/Mo_2_N-NrGO and the performance of AEMWE cell (Pt/Mo_2_N-NrGO || NiFe LDH) with other Pt-based electrocatalysts in 1.0 M KOH

| Catalyst | Tafel slope (mV dec^-1^) | η_10_^a)^  (mV) | TOF at 0.1 V (s^-1^) | AEMWE Cell | Cell voltage at 1A cm^-2^ (V) | AEMWE stability | References |
| --- | --- | --- | --- | --- | --- | --- | --- |
| **AlO@Mo_2_N-NrGO** | **31.0** | **11.0** | **6.3** | **Pt/Mo_2_N-NrGO \|\| NiFe LDH** | **1.87** | **95.24%@500h at 1.5 A·cm^-2^** | **This work** |
| Pt/CoV-LDHs | 37 | 37.0 | 1.3 | - | - | - | [S4] |
| Pt-AD-NiOOH | 57.0 | 13.0 | - | - | - | - | [S5] |
| Pt–Ru/RuO_2_ | 50.7 | 18.0 | - | Pt–Ru/RuO_2_// NiFe LDH | 1.90 | - | [S6] |
| Pt–Ni_4_Mo/CNT | 37.4 | 18.6 | 6.24 | - | - | - | [S7] |
| Pt/CeOx/C | 50 | 19 | - | - | - | - | [S8] |
| 1%Pt-NiCoP@Mxene | 38.6 | 26.5 | 3.6 | 1%Pt-NiCoP@Mxene \|\| NiFeLDH | 1.95 | 96%@500h at 0.5 A·cm^-2^ | [S9] |
| Pt_1_/CeOx | 34 | 37 | - | - | - | - | [S10] |
| 2D-Pt/LDH | 32.2 | 25 | - | - | - | - | [S11] |
| Pt@CoS | 32.0 | 28 | 0.131 | - | - | - | [S12] |
| Pt/MgO | 39 | 39 | - | - | - | - | [S13] |
| Pt-AC/Cr−N−C | 32 | 19 | 4.1 | Pt-AC/Cr−N−C \|\| NiFeLDH | 1.9 | 9 mV@100h at 0.5 A·cm^-2^ | [S14] |
| Pt1.5Rh1.5Tm | 33.8 | 20 | - | - | - | - | [S15] |
| Pt-a/c-CoN | 33.6 | 19 | - | - | - | - | [S16] |
| HEA-PdPtRhIrCu | 37 | 15 | 3.16 |  |  |  | [S17] |
| Pt_SA_–Mn3O4 | 54 | 24 |  |  |  |  | [S18] |
| CC@MoS2/Pt1,n | 40.8 | 35.6 | 1.27 |  |  |  | [S19] |
| PtRu NCs/BP | 43 | 22 |  |  |  |  | [S20] |
| Pt–C core-shell@h-MoS2/GNF | 32 | 30 | - | Pt–C core-shell@h-MoS2/GNF \|\| NiCo2O4 | 2.08 | 14h at 0.1 A·cm^-2^ | [S21] |
| Pt@Co-NPC-800 | 42.72 | 34 |  | Pt@Co-NPC-800 \|\| NiCo2O4 | 2.2 | 20 h at 0.3 A·cm^-2^ | [S22] |
| Pd44Pt30Ir26ASNSs/C | 36.1 | 17 |  | Pd44Pt30Ir26ASNSs/C \|\| Pd44Pt30Ir26ASNSs/C | 1.8 V (400 mA cm^-2^) | 40 h at 0.5 A·cm^-2^ | [S23] |
| Turing PtNiNb | 30 | 27.2 | 27.5 | Turing PtNiNb \|\| PtIr | 1.98 | 500 h at 1 A·cm^-2^ | [S24] |
| Cl-Pt/LDH | 75.6 | 25.2 | 30.3 | Cl-Pt/LDH \|\| Ir/C | 1.99 | 32 mV@20h at 1 A·cm^-2^ | [S25] |
| aPt/pNi-NOMC | 40 | 65 | - | aPt/pNi-NOMC \|\| Co3O4 | 1.9 | 200 h at 0.5 A·cm^-2^ | [S26] |

**Supplementary References**

1. F.-Y. Yu, Z.-L. Lang, Y.-J. Zhou, K. Feng, H.-Q. Tan et al., Revealing hydrogen evolution performance of single-atom platinum electrocatalyst with polyoxometalate molecular models. ACS Energy Lett. **6**(11), 4055–4062 (2021). <https://doi.org/10.1021/acsenergylett.1c01911>
2. E. Taibi, H. Blanco, R. Miranda, M. Carmo. *Hydrogen cost reduction: Scaling up electrolysers to meet the 1.5 oc climate goal*. (IRENA; Abu Dhabi, 2020).
3. B. Lee, H.-S. Cho, H. Kim, D. Lim, W. Cho et al., Integrative techno-economic and environmental assessment for green H_2_ production by alkaline water electrolysis based on experimental data. J. Environ. Chem. Eng. **9**(6), 106349 (2021). <https://doi.org/10.1016/j.jece.2021.106349>
4. X. Li, Y. Yan, Y. Yao, Y. Liu, Three-in-one tandem catalysis for alkaline hydrogen evolution reaction on Pt/CoV-LDHs. Chem. Eng. J. **489**, 151237 (2024). <https://doi.org/10.1016/j.cej.2024.151237>
5. Z. Lin, Z. Wang, J. Gong, T. Jin, S. Shen et al., Reversed spillover effect activated by Pt atom dimers boosts alkaline hydrogen evolution reaction. Adv. Funct. Mater. **33**(45), 2307510 (2023). <https://doi.org/10.1002/adfm.202307510>
6. Y. Zhu, M. Klingenhof, C. Gao, T. Koketsu, G. Weiser et al., Facilitating alkaline hydrogen evolution reaction on the hetero-interfaced Ru/RuO_2_ through Pt single atoms doping. Nat. Commun. **15**(1), 1447 (2024). <https://doi.org/10.1038/s41467-024-45654-9>
7. J. Liu, Z. Wang, X. Wu, D. Zhang, Y. Zhang et al., Pt doping and strong metal–support interaction as a strategy for NiMo-based electrocatalysts to boost the hydrogen evolution reaction in alkaline solution. J. Mater. Chem. A **10**(29), 15395–15401 (2022). <https://doi.org/10.1039/D2TA03934F>
8. S.-W. Yu, S. Kwon, Y. Chen, Z. Xie, X. Lu et al., Construction of a Pt-CeOx interface for the electrocatalytic hydrogen evolution reaction. Adv. Funct. Mater. **34**(38), 2402966 (2024). <https://doi.org/10.1002/adfm.202402966>
9. H.-J. Niu, C. Huang, T. Sun, Z. Fang, X. Ke et al., Enhancing Ni/co activity by neighboring Pt atoms in NiCoP/MXene electrocatalyst for alkaline hydrogen evolution. Angew. Chem. Int. Ed **63**(20), e202401819 (2024). <https://doi.org/10.1002/anie.202401819>
10. V. Dao, G. Di Liberto, S. Yadav, P. Uthirakumar, K. Chen et al., Pt single atoms supported on defect ceria as an active and stable dual-site catalyst for alkaline hydrogen evolution. Nano Lett. **24**(4), 1261–1267 (2024). <https://doi.org/10.1021/acs.nanolett.3c04237>
11. Y.R. Hong, S. Dutta, S.W. Jang, O.F. Ngome Okello, H. Im et al., Crystal facet-manipulated 2D Pt nanodendrites to achieve an intimate heterointerface for hydrogen evolution reactions. J. Am. Chem. Soc. **144**(20), 9033–9043 (2022). <https://doi.org/10.1021/jacs.2c01589>
12. A. Mosallanezhad, C. Wei, P. Ahmadian Koudakan, Y. Fang, S. Niu et al., Interfacial synergies between single-atomic Pt and CoS for enhancing hydrogen evolution reaction catalysis. Appl. Catal. B Environ. **315**, 121534 (2022). <https://doi.org/10.1016/j.apcatb.2022.121534>
13. H. Tan, B. Tang, Y. Lu, Q. Ji, L. Lv et al., Engineering a local acid-like environment in alkaline medium for efficient hydrogen evolution reaction. Nat. Commun. **13**(1), 2024 (2022). <https://doi.org/10.1038/s41467-022-29710-w>
14. L. Zeng, Z. Zhao, Q. Huang, C. Zhou, W. Chen et al., Single-atom Cr-N_4_ sites with high oxophilicity interfaced with Pt atomic clusters for practical alkaline hydrogen evolution catalysis. J. Am. Chem. Soc. **145**(39), 21432–21441 (2023). <https://doi.org/10.1021/jacs.3c06863>
15. Q. Li, B. Zhang, C. Sun, X. Sun, Z. Li et al., Enhanced alkaline hydrogen evolution reaction *via* electronic structure regulation: activating PtRh with rare earth Tm alloying. Small **20**(32), 2400662 (2024). <https://doi.org/10.1002/smll.202400662>
16. Y. Li, X. Zhang, L. Liu, H. Sheng, C. Li et al., Ultra-low Pt doping and Pt-Ni pair sites in amorphous/crystalline interfacial electrocatalyst enable efficient alkaline hydrogen evolution. Small **19**(23), e2300368 (2023). <https://doi.org/10.1002/smll.202300368>
17. Q. Mao, X. Mu, K. Deng, H. Yu, Z. Wang et al., Multisite synergism-induced electron regulation of high-entropy alloy metallene for boosting alkaline hydrogen evolution reaction. Adv. Funct. Mater. **33**(42), 2304963 (2023). <https://doi.org/10.1002/adfm.202304963>
18. J. Wei, K. Xiao, Y. Chen, X.-P. Guo, B. Huang et al., *In situ* precise anchoring of Pt single atoms in spinel Mn_3_O_4_ for a highly efficient hydrogen evolution reaction. Energy Environ. Sci. **15**(11), 4592–4600 (2022). <https://doi.org/10.1039/D2EE02151J>
19. X. Wang, X. Zhang, Y. Xu, H. Song, X. Min et al., Heterojunction Mo-based binary and ternary nitride catalysts with Pt-like activity for the hydrogen evolution reaction. Chem. Eng. J. **470**, 144370 (2023). <https://doi.org/10.1016/j.cej.2023.144370>
20. Y. Li, W. Pei, J. He, K. Liu, W. Qi et al., Hybrids of PtRu nanoclusters and black phosphorus nanosheets for highly efficient alkaline hydrogen evolution reaction. ACS Catal. **9**(12), 10870–10875 (2019). <https://doi.org/10.1021/acscatal.9b03506>
21. S. Ramakrishnan, S. Vijayapradeep, S.C. Selvaraj, J. Huang, S.C. Karthikeyan et al., An efficient cathode electrocatalyst for anion exchange membrane water electrolyzer. Carbon **220**, 118816 (2024). <https://doi.org/10.1016/j.carbon.2024.118816>
22. M.R. Subramaniam, S. Ramakrishnan, S. Sidra, S.C. Karthikeyan, S. Vijayapradeep et al., Carbon core–shell Pt nanoparticle embedded porphyrin Co-MOF derived N-doped porous carbon for the alkaline AEM water electrolyzer application. J. Mater. Chem. A **12**(10), 5967–5979 (2024). <https://doi.org/10.1039/D3TA06745A>
23. Z. Lyu, X. Zhang, X. Liao, K. Liu, H. Huang et al., Two-dimensionally assembled Pd–Pt–Ir supernanosheets with subnanometer interlayer spacings toward high-efficiency and durable water splitting. ACS Catal. **12**(9), 5305–5315 (2022). <https://doi.org/10.1021/acscatal.2c00859>
24. J. Gu, L. Li, Y. Xie, B. Chen, F. Tian et al., Turing structuring with multiple nanotwins to engineer efficient and stable catalysts for hydrogen evolution reaction. Nat. Commun. **14**(1), 5389 (2023). <https://doi.org/10.1038/s41467-023-40972-w>
25. T. Zhang, J. Jin, J. Chen, Y. Fang, X. Han et al., Pinpointing the axial ligand effect on platinum single-atom-catalyst towards efficient alkaline hydrogen evolution reaction. Nat. Commun. **13**(1), 6875 (2022). <https://doi.org/10.1038/s41467-022-34619-5>
26. W.-G. Lim, H.N. Truong, J.-Y. Jeong, D. Kim, L.S. Oh et al., Toward feasible single atom-based hydrogen evolution electrocatalysts *via* artificial ensemble sites for anion exchange membrane water electrolyzer. Appl. Catal. B Environ. **343**, 123568 (2024). <https://doi.org/10.1016/j.apcatb.2023.123568>
